# Evaluating implementation of the World Health Organization’s Strategic Approach to strengthening sexual and reproductive health policies and programs to address unintended pregnancy and unsafe abortion

**DOI:** 10.1186/s12978-017-0405-3

**Published:** 2017-11-21

**Authors:** Shusmita Rashid, Julia E. Moore, Caitlyn Timmings, Joshua P. Vogel, Bela Ganatra, Dina N. Khan, Radha Sayal, A. Metin Gülmezoglu, Sharon E. Straus

**Affiliations:** 1 University of Toronto, 1 King’s College Circle, Medical Sciences Building, Toronto, ON M5S 1A8 Canada; 2 UNDP/UNFPA/UNICEF/WHO/World Bank Special Programme of Research, Development and Research Training in Human Reproduction (HRP), Department of Reproductive Health and Research, World Health Organization, Headquarters, 20 Avenue Appia, 1211 Geneva, Switzerland

**Keywords:** Strategic Approach, Sexual reproductive health, Implementation, Process evaluation, Contextual factors, Unsafe abortion, Unintended pregnancy, Social ecological model

## Abstract

**Background:**

We conducted a process evaluation to assess how the World Health Organization’s (WHO) *Strategic Approach to strengthening sexual and reproductive health policies and programs* (“the SA”) was used in 15 countries that requested WHO’s technical support in addressing unintended pregnancy and unsafe abortion. The SA is a three-stage planning, policy, and program implementation process. We used the social ecological model (SEM) to analyze the contextual factors that influenced SA implementation.

**Methods:**

We used a two-phased sequential approach to data collection and analysis. In Phase A, we conducted a document and literature review and synthesized data thematically. In Phase B, we conducted interviews with stakeholders who used the SA in the countries of interest. We used a qualitative method triangulation technique to analyze and combine data from both phases to understand how the SA was implemented in each country.

**Results:**

Data from 145 documents and 19 interviews described the SA process and activities in each country. All 15 countries completed Stage 1 activities. The activities of Stage 1 determined activities in subsequent stages and varied across countries. Following Stage 1, some countries focused on reforming policies to improve access to sexual and reproductive health (SRH) services whereas others focused on improving provider-level capacity to enhance SRH service quality and improving community-level SRH education. We identified factors across SEM levels that affected SA implementation, including individual- and community-level perceptions of using the SA and the recommendations that emerged from its use, organizational capacity to conduct SA activities, and how well these activities aligned with the existing political climate. Stakeholders perceived SA implementation to be country-driven and systematic in bringing attention to important SRH issues in their countries.

**Conclusion:**

We identified key success factors for influencing the individual, organization, and system change required for implementing the SA. These include sustaining stakeholder engagement for all SA stages, monitoring and reporting on activities, and leveraging activities and outputs from each SA stage to obtain technical and financial support for subsequent stages. Results may be used to optimize ongoing implementation efforts to improve access to and the quality of SRH services.

**Electronic supplementary material:**

The online version of this article (10.1186/s12978-017-0405-3) contains supplementary material, which is available to authorized users.

## Plain English summary

We evaluated how the World Health Organization’s planning, policy, and program implementation process, (called the *Strategic Approach to strengthening sexual and reproductive health policies and programs* or “*the SA*”), was used in 15 countries to address unintended pregnancy and unsafe abortion. SA implementation was influenced by individual-, community-, organizational-, and policy-level factors. Stakeholders perceived SA implementation to be country-driven and systematic in bringing attention to important sexual and reproductive health issues in their countries. Key success factors for implementing the SA include ensuring stakeholder engagement during all stages of SA activities, monitoring activities, and using outputs from each SA stage to gain support for subsequent stages. Findings from this study can guide efforts in countries that are currently using the SA or conducting similar activities to improve sexual and reproductive health services.

## Background

Of the 210 million pregnancies that occur globally each year, it is estimated that 80 million are unintended [1]. In low- and middle-income countries, 24% of pregnancies ended in abortion in 2010 to 2014 [2]. Women are more likely to resort to an unsafe abortion when provisions for safe abortions are restricted, unavailable, or inaccessible [3]. Recent studies estimate that 8–18% of maternal deaths worldwide are due to unsafe abortion, and the estimated number of abortion-related maternal deaths in 2014 ranged from 22,500 to 44,000 [4–6]. Almost all abortion-related deaths occur in low- and middle-income countries, and estimates for 2012 indicate that 6.9 million women in the developing world were treated for complications from unsafe abortion [7].

Reducing the incidence and consequences of unsafe abortion are essential for achieving United Nations Sustainable Development Goal 3 — to ensure universal access to sexual and reproductive health (SRH) care and reduce global maternal death rates. Over the past 15 years, SRH decision makers in several countries have worked in collaboration with non-governmental organizations (NGOs) and international agencies to adopt the World Health Organization’s (WHO) Strategic Approach to strengthening SRH policies and programs (herein referred to as “the SA”). The SA is a planning, policy, and program implementation process that countries can use to assess their SRH needs and strengthen policies and programs to address these needs. Since being field tested in 1993 as a systematic approach to contraceptive introduction, the SA has been used to address a broad range of SRH issues (particularly reproductive tract infections, HIV/AIDs, maternal health, and family planning). From 1997 to 2014, 15 countries (i.e., Vietnam, Romania, Bangladesh, Mongolia, Ghana, Moldova, Macedonia, Ukraine, Zambia, Guinea, Malawi, Russian Federation, Senegal, Kyrgyzstan, and Sierra Leone) requested technical support from the WHO to use the SA to address unintended pregnancy and unsafe abortion. The current study is a process evaluation of how the SA was applied in these 15 countries. This evaluation was not intended to measure the impact of the SA on reducing unsafe abortion in these countries.

**Fig. 1:**
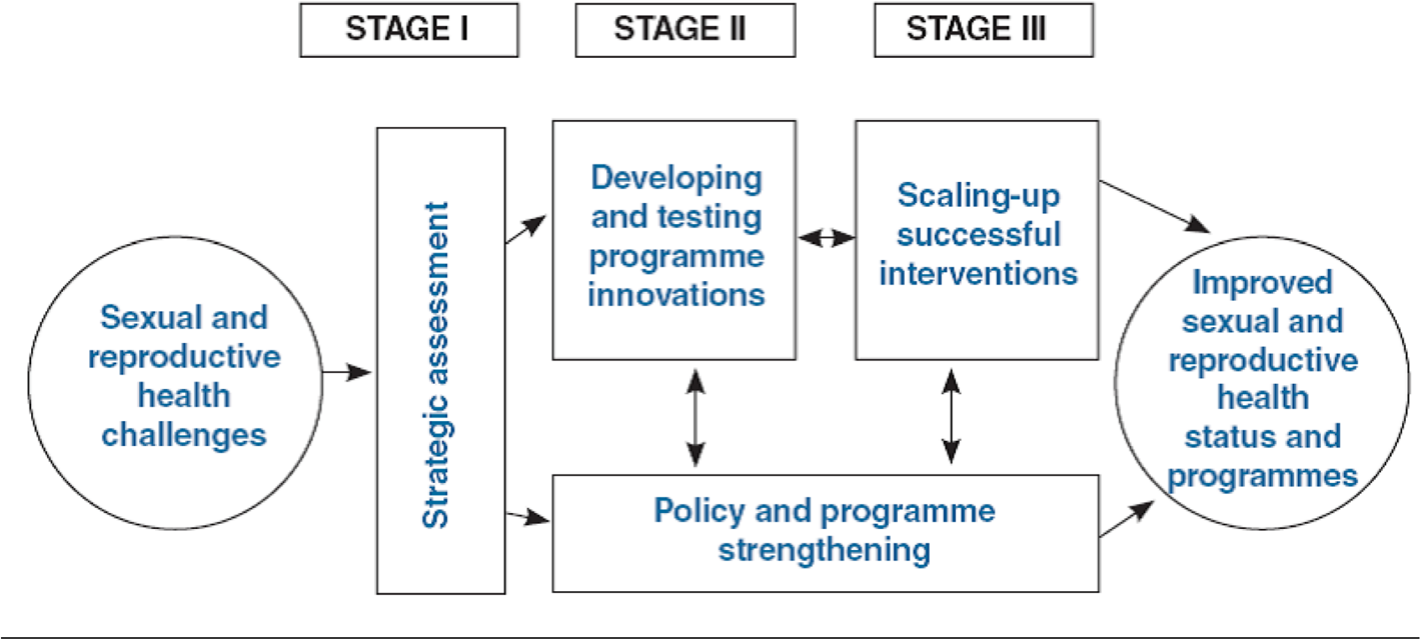
The SA implementation process [8]

The SA is guided by public health, social and management sciences, and the International Conference on Population and Development (ICPD) principles; it is a three-staged implementation process that uses a systems framework, a participatory process emphasizing country ownership, and a reproductive health philosophy focusing on improving equitable access to and quality of care [8]. The SA is composed of the following stages (Fig. 1):

- **Stage 1**: Conducting a field-based strategic assessment to identify and prioritize SRH needs and generate consensus recommendations for addressing these needs;
- **Stage 2**: Developing and pilot testing innovations (e.g., policy, programs, and services) on a limited scale at different levels of the health system and evaluating these interventions to determine if implementation is feasible, acceptable, effective, and sustainable in the particular context; and
- **Stage 3**: Scaling up to expand access beyond pilot sites and strengthening health system capacity to sustain the provision of programs and services.

### Evaluating the “process” of implementing the SA

There is a gap in research on understanding the processes and mechanisms by which complex interventions are implemented in practice [9–11]. Many SRH programs report on program impacts without describing how, when, where, and under what conditions programs were developed and implemented [12]. As a result, it is difficult to replicate programs that are successful in achieving intended outcomes. For unsuccessful programs, it is unclear whether the program is ineffective, whether it was limited by contextual factors, or whether it was not implemented well [10]. Process evaluations can help to unpack the “black box” of implementation by providing insight into how program components interact with factors in the implementation context that help or hinder implementation processes [11, 12].

We conducted a process evaluation guided by the following objectives:

- To understand how the SA was applied in each of the 15 countries and analyze the processes and outputs of each stage of the SA;
- To identify and analyze contextual factors that influenced implementation of the SA using the Social Ecological Model (SEM; Additional file 1) as an analytical framework to examine health system dynamics across countries and time; and
- To identify key success factors for implementing the SA to inform current and future implementation efforts.

## Methods

**Fig. 2:**
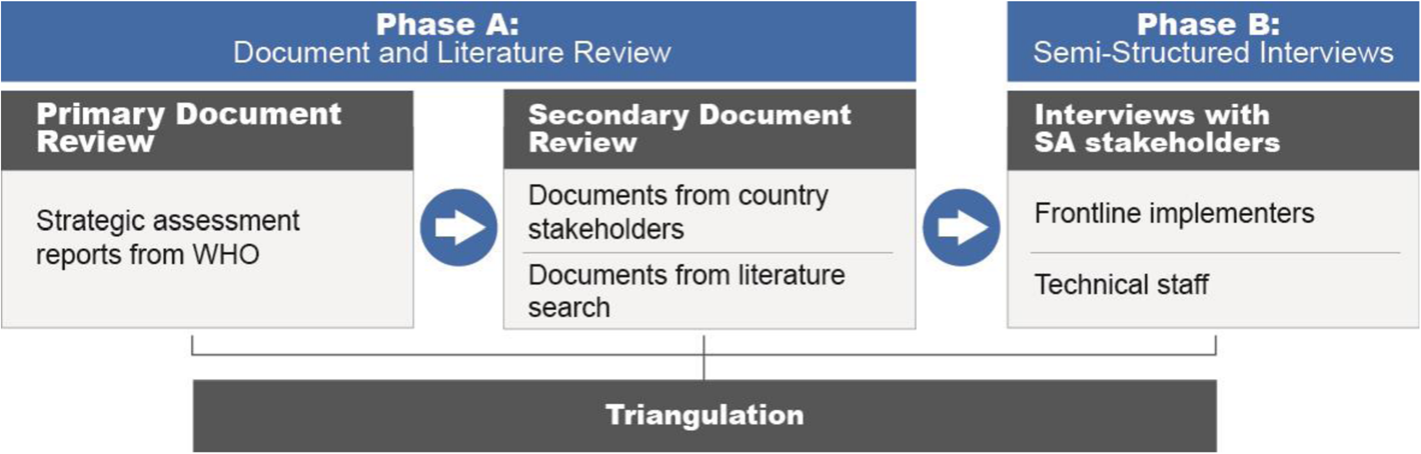
Overview of data collection and analysis

We used a two-phased qualitative sequential approach (Fig. 2).

### Phase A: document and literature review

The primary document review phase (conducted between April and December 2015) was designed to collect and analyze the strategic assessment reports for all 15 countries. We then identified and analyzed secondary documents (e.g., country background papers; historical documents; national standards, policies, and guidelines for abortion care; donor reports; and information educational materials).

A librarian conducted a literature search of databases (e.g., MEDLINE and EMBASE) and grey literature in July 2015 to identify English-language articles (published between 1946 and 2015) on the SA and reproductive health issues in the countries of interest. Search terms consisted of keywords, such as “World Health Organization”, “strategic approach”, “abortion”, “birth control”, “contraception”, “fertility control”, “family planning”, “unwanted pregnancy”, and “unplanned pregnancy”, (Additional file 2). Two reviewers independently screened the titles and abstracts of records to select articles for full-text review. Articles were included for full-text review if they explicitly mentioned the WHO’s SA recommendations and/or described SA initiatives being implemented at the national or local level in any of the 15 countries of interest. Articles were excluded if they did not directly mention the WHO’s SA or described initiatives outside of the countries of interest. Inter-rater reliability was calculated to assess the degree to which the two reviewers consistently agreed on which articles were eligible for full-text review. The calculated Kappa coefficient, *k* = 0.82, indicated substantial inter-rater agreement [13]; discrepancies were reconciled through discussion. Full-text versions of the selected articles were then retrieved and reviewed by two independent reviewers to determine eligibility.

#### Data abstraction

Primary and secondary documents were organized by country or document type. Data were abstracted from the documents using a standardized template (Additional file 3). The data abstraction template was piloted by two reviewers, who independently used the template to review a primary document from one country selected at random. The reviewers then met to deliberate and refine the template. Subsequently, 20% of the primary documents were randomly selected, double coded, and analyzed for reliability across two independent reviewers (*k* = 0.66). Reviewers reconciled discrepancies through discussion and further revised the data abstraction template to create a final version. Reviewers then used the final template to independently abstract data from the remaining documents.

#### Data analysis

Two analysts reviewed abstracted data from the documents. Following familiarization with the data, analysts generated initial codes, identified themes among the list of codes, and developed a thematic framework of analysis [14]. Analysts then reviewed and synthesized abstracted data according to the major themes.

### Phase B: semi-structured interviews

Interviews were conducted between September 2015 and January 2016 to explore perceptions of two stakeholder groups: (1) frontline implementers who used the SA for policy and program strengthening in their countries and (2) technical staff who had a role in development, operations, or technical support of the SA activities in these countries (Fig. 2).

Stakeholders were identified and contacted by the WHO and introduced to the research staff. The research staff asked country stakeholders to provide additional documents and names of potential stakeholders who could be recruited for interviews through purposive and snowball sampling. Interviews were 45–60 min in length and conducted in English by experienced interviewers via telephone or in person using semi-structured interview guides (Additional file 4). Interview guide development was informed by research on factors that affect the implementation process [15]. Interviews were audio recorded and transcribed verbatim and de-identified prior to analysis.

#### Data analysis

The SEM, a theory-based framework [16–18], was used to understand the interplay of factors and conditions under which the SA has been used and the processes involved with implementing the SA over time (1997–present) and in different contexts (15 countries). An overview of the SEM and other relevant frameworks is provided in Additional file 1.

Two qualitative analysts independently familiarized themselves with the data by reviewing interview field notes and transcripts to develop an initial list of codes and themes. Analysts then compared their initial list of codes and developed a thematic framework of analysis (i.e., the codebook), which was subsequently applied to the data (Additional file 5). This framework was piloted with two transcripts and further refined to fit the data. The framework was adapted throughout the coding process to capture emergent themes [14]. A modified audit consensus coding approach [19] was used whereby interview transcripts were divided into six groups and coded in sequential rounds. Analysts independently applied the coding framework to interview transcripts and at the end of each round, inter-rater reliability between analysts was determined using the Kappa coefficient. Analysts discussed discrepancies and made changes to the coding file until a Kappa coefficient ≥ 0.6 [20] was achieved for each individual code/transcript combination. The number of discrepancies between coders decreased after the second round of coding. For the remaining transcripts, the primary analyst coded all transcripts and the secondary analyst audited one randomly selected transcript per round. Once all data were coded, both analysts met to discuss the emergent themes and to chart data according to theme and country using the framework matrices tool in NVivo 10.

### Triangulation

A multi-source (i.e., document and literature review and interviews) qualitative method triangulation technique [21] was applied to analyze the data. Data collected from all sources were combined through a matrix where data were mapped according to country, SEM, and stage of the SA. This method allowed analysts to consider data from the document and literature review and interviews collectively to better understand how the SA was implemented in each country.

#### Ethics, consent, and permissions

Ethics approval was obtained from the University of Toronto Health Sciences Research Ethics Board (REB #31181), and verbal informed consent was obtained from all participants. Data were de-identified prior to analysis.

## Results

The number of data sources (i.e., document and literature review from Phase A and semi-structured interviews from Phase B) per country varied (Additional file 6), with the highest proportion of data coming from Malawi (*n* = 21) and Ghana (*n* = 18), followed by Macedonia (*n* = 16) and Zambia (*n* = 16). Romania (*n* = 4) had the lowest number of available data sources. A breakdown of the results from Phase A and Phase B is provided below.

### Phase A: document and literature review

**Fig. 3:**
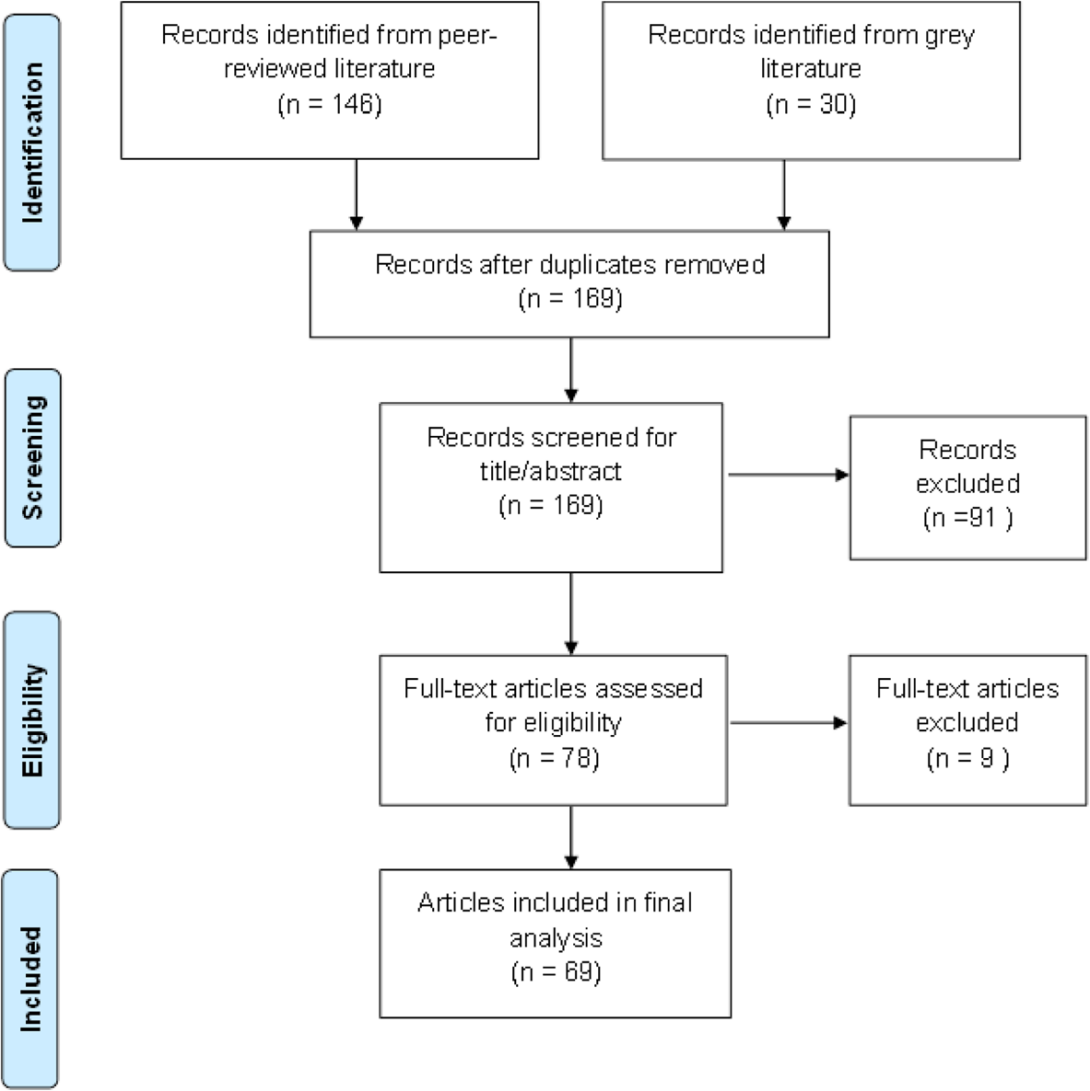
Study flow for literature search

For the primary document review, we analyzed 15 strategic assessment reports. For the secondary document review, we analyzed 61 documents from country stakeholders and 69 documents from the literature search. The literature search (Fig. 3) yielded 169 records after duplicates were removed. After screening for title and abstract (Stage 1) and conducting full-text screening of articles (Stage 2), 69 documents were included in the final analysis. Additional file 7 provides the complete list of citations.

The 69 documents selected from the literature search described a wide range of SRH policies, programs, and practices. Of the 44 peer-reviewed articles, 13 articles examined abortion methods and practices, 11 articles evaluated programs to improve access to and quality of SRH services, eight articles reported the incidence of abortion and/or abortion-related deaths, four articles were related to reproductive rights and policy reform, three articles described social factors and attitudes towards contraceptives and abortions, three articles examined specific strategies and tools to improve the quality of SRH services, and two articles offered guidance on providing high-quality SRH services. The 25 grey literature documents included progress reports, highlights reports, as well as biennial and annual technical reports from WHO’s Department of Reproductive Health and Research.

### Phase B: semi-structured interviews

We completed 19 interviews with a response rate of 83%: 11 with technical staff and eight with frontline implementers.

### Overview of SA implementation and factors affecting progression through the SA stages

**Fig. 4:**
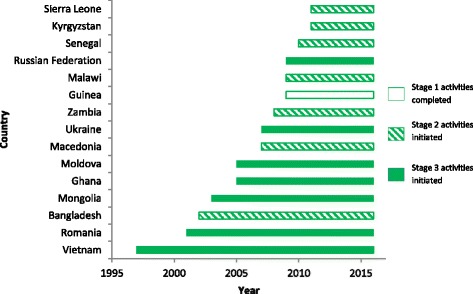
Progression through SA stages to date by country

Figure 4 presents the timeframe during which the SA was implemented in each country. All 15 countries completed Stage 1 activities. All except one country (Guinea) initiated Stage 2 activities based on Stage 1 outputs/recommendations. Seven countries (i.e., Ghana, Moldova, Mongolia, Romania, Russian Federation, Ukraine, and Vietnam) were perceived to have simultaneously engaged in Stage 2 activities (i.e., piloting certain innovations) and Stage 3 activities (i.e., scaling up other innovations from Stage 2). Interview participants could not confirm the status of activities (i.e., whether these activities were ongoing or completed) in the countries that had initiated Stage 3. Examples of common country-specific activities by stage are provided in Table 1.

**Table 1:** Examples of SA activities by country

Country	Stage 1		Examples of Stage 2 and 3 activities
Bangladesh	Year initiated	2002	• Developed menstrual regulation guidelines. • Used information, education, and communication materials to disseminate information through fieldworkers. • Focused on policy reform and securing funding for menstrual regulation kits and commodities.
	Assessment team	11 members.	
	Stakeholders involved	Physicians and social scientists.	
	Technical support	WHO	
	Assessment sites	Fieldwork was conducted in 5 districts and at the central level.	
Ghana	Year initiated	2005	• Developed and disseminated standards and guidelines and trained mid-level HCPs to increase quality and availability of services. • Registered Medabon®, a co-packaged mifepristone-and-misoprostol product for medical abortion, which is approved for use by physicians in both public and private facilities. • Created a fixed price for abortion services in public facilities with a fee-sharing provision for abortion providers to discourage clandestine provision of services. • Conducted sensitization training for HCPs, members of the media, lawyers, police officers, and community leaders on legal indications for abortion, the incidence and impact of unsafe abortion, and ways to prevent it. • Conducted a nationwide maternal health study with emphasis on abortion. • Updated national monitoring system to improve the monitoring and evaluation on CAC. • Initiated scale up of CAC and family planning services, mainly through international partners, although some districts and regions raised their own funds.
	Assessment team	17 members	
	Stakeholders involved	Policymakers, program managers, HCPs, and reproductive rights and women’s health advocates.	
	Technical support	Ipas and WHO	
	Assessment sites	Fieldwork was conducted in 6 administrative regions.	
Guinea	Year initiated	2009	• Country stakeholders were unable to secure funding or technical support to move beyond Stage 1.
	Assessment team	18 members	
	Stakeholders involved	Health professionals and representatives from NGOs, government agencies, research centres, and community organizations.	
	Technical support	WHO	
	Assessment sites	Fieldwork was conducted in 4 regions and the country’s capital.	
Kyrgyzstan	Year initiated	2011	• Developed new health strategy and provided training on new health strategy. • Increased access to family planning services and contraception. • Improved sexuality education. • Conducted medical abortion operations research study and trained midwives to improve access to medical abortion in rural areas.
	Assessment team	14 members	
	Stakeholders involved	Clinical and research experts.	
	Technical support	UNFPA, UNICEF, and WHO	
	Assessment sites	Fieldwork was conducted in 3 regions.	
Macedonia	Year initiated	2007	• Developed national strategy for sexual and reproductive health, which was adopted by the MOH in 2011. • Allocated funding in the national program budget for the operation of a youth counselling centre that provides free contraceptives and education materials.
	Assessment team	13 members	
	Stakeholders involved	MOH and government agencies, community and clinical organizations, and NGOs.	
	Technical support	UNDP, UNFPA, and WHO	
	Assessment sites	Fieldwork was conducted in 6 regions.	
Malawi	Year initiated	2009	• Conducted study to understand complications of unsafe abortion and cost to the health system. • Focused on increasing provider-level capacity and facility-level equipment to improve PAC. • Formed a local civil society coalition to advocate for legal reform.
	Assessment team	24 members	
	Stakeholders involved	Government agencies, human rights groups, and NGOs.	
	Technical support	Ipas and WHO	
	Assessment sites	Fieldwork was conducted in 10 districts.	
Moldova	Year initiated	2005	• Developed standards and guidelines for safe abortion services and trained HCPs. • Increased access to contraceptives for youth and socially vulnerable groups through insurance system coverage. • Piloted CAC at 4 model centres and subsequently included 2 more model centres.
	Assessment team	23 members	
	Stakeholders involved	MOH, clinical organizations, legal organization, researchers, NGOs, HCPs, and mass media.	
	Technical support	East European Institute of Reproductive Health in Romania, Ipas, and WHO.	
	Assessment sites	Fieldwork was conducted in 9 administrative units and 2 municipalities.	
Mongolia	Year initiated	2003	• Developed national standards and guidelines for abortion and the national pre-service training curriculum was harmonized with the new guidelines. • Registered Mifepristone and Misoprostol for first and second trimester abortion and included these drugs in the National Drugs Registry in 2006. • Established 3 model CAC units to provide high quality services and used these units as training centres for HCPs. • Focused on improving national facility infrastructure and upgrading diagnostic and treatment centres.
	Assessment team	19 members	
	Stakeholders involved	Public health institute, research centres, youth organizations, and HCPs.	
	Technical support	WHO, Population Council (Bangkok), Ipas	
	Assessment sites	Fieldwork was conducted in 6 provinces and the nation’s capital.	
Romania	Year initiated	2001	• Developed standards and guidelines and improved infrastructure in several hospitals to provide high-quality abortion services. • Improved access to contraceptive services by making them available from family physicians (not just gynecologists as was previously done). • Pilot-tested free contraceptives intervention in 3 rural districts and scaled up to 42 districts to make contraceptives available to vulnerable groups of the population.
	Assessment team	19 members	
	Stakeholders involved	MOH, clinical organizations, government agencies, NGOs, and HCPs.	
	Technical support	WHO and Ipas	
	Assessment sites	Fieldwork was conducted in 8 administrative units and the county’s capital.	
Russian Federation	Year initiated	2009	• Revised regulatory documents and developed national guidelines, standards, and protocols according to WHO recommendations. • Trained obstetricians and gynecologists on revised guidelines through post-graduate education. • Conducted local operations research on safe practices of induced abortion in the first trimester. • Increased accessibility to abortion services.
	Assessment team	25 members	
	Stakeholders involved	Researchers, community organizations, and health care professionals.	
	Technical support	WHO	
	Assessment sites	Fieldwork was conducted in 3 regions	
Senegal	Year initiated	2010	• Formed an advocacy task force, which conducted awareness-raising workshops with parliamentarians, religious leaders, journalists, and civil-society groups. • Discussed a draft bill that includes all indications for abortion stipulated by the Maputo Plan of Action during a workshop in June 2011. • Developed a national dialogue about unsafe abortion and the need for legislative changes and country stakeholders have been advocating for these changes. • Initiated plans to improve sexual and reproductive health education, family planning, and PAC services.
	Assessment team	28 members	
	Stakeholders involved	MOH, civil society, government agencies, and NGOs.	
	Technical support	Ipas	
	Assessment sites	Fieldwork was conducted in 10 regions.	
Sierra Leone	Year initiated	2011	• Ongoing efforts to revise the abortion law resulted in the country’s members of parliament voting unanimously in favour of legislation that would legalize abortion at up to 12 weeks of pregnancy in December 2015. However, amidst religious protests, the country’s president declined to sign the bill. In February 2016, rights groups urged the president to give the bill assent. The bill has been referred to the constitutional review committee, which is currently reviewing the country’s constitution.
	Assessment team	27 members	
	Stakeholders involved	MOH, health professionals, NGO, and legal professionals.	
	Technical support	Ipas and WHO	
	Assessment sites	Fieldwork was conducted in 12 health districts.	
Ukraine	Year initiated	2007	• Implemented Comprehensive Care for Unwanted Pregnancies project (CCUP), which resulted in 5 new model clinics supported with capacity building activities on CCUP provision. • Implemented new training curriculum on CCUP for obstetricians and gynecologists. • Monitored and evaluated CCUP services in pilot regions. • Developed scaling-up strategy for CCUP, which has been distributed to all the regions of Ukraine for implementation.
	Assessment team	32 members	
	Stakeholders involved	MOH, government agencies, higher educational establishment, and professional associations.	
	Technical support	WHO and Ipas	
	Assessment sites	Fieldwork was conducted in 2 regions.	
Vietnam	Year initiated	1997	• Formed National Technical Working Group on Abortion to finalize national technical guidelines for abortion services and included abortion-related policy recommendations in a national reproductive health strategy. • Conducted the Comprehensive Abortion Care project from 2001 to 2009 and set up national abortion care guidelines for all health care levels to use modern abortion techniques.
	Assessment team	12 members	
	Stakeholders involved	MOH, clinical and community organizations, and physicians.	
	Technical support	WHO	
	Assessment sites	Fieldwork was conducted in 6 provinces.	
Zambia	Year initiated	2008	• Developed and disseminated CAC standards and guidelines to increase quality and availability of services. • Conducted action research to introduce medical abortion drugs in 20 health facilities. • Registered Medabon® and the government has begun to allocate funds for purchasing medicines/equipment for abortion services. • Conducted a pilot program for distributing contraceptive injections through community-based workers.
	Assessment team	17 members	
	Stakeholders involved	Academics, program managers, HCPs, researchers, and women’s health advocates.	
	Technical support	Ipas and WHO	
	Assessment sites	Fieldwork was conducted in 5 provinces.	

A number of factors were perceived to have influenced the implementation of the SA overall and progression through the SA stages.

#### Individual perceptions of using the SA to address SRH issues

Some senior-level stakeholders believed that they understood the scope of unintended pregnancy and unsafe abortion in their respective countries and felt that they did not need to collect additional data through Stage 1 assessment activities. Others believed that qualitative assessment activities (i.e., interviews and discussions) were not appropriate in their context because no one would openly discuss a stigmatized topic, such as abortion, and quantitative methods were preferred to understand the magnitude of the problem. Participation in global SRH conferences was perceived to have stimulated stakeholders’ (e.g., from Ghana, Malawi, and Zambia) interests in using the SA. Similarly, Ministry of Health (MOH) and WHO endorsement of the SA provided credibility to the process and influenced country stakeholders’ decisions to use the SA.

#### In-country technical capacity to develop, pilot test, and evaluate interventions

In some countries (e.g., Bangladesh and Ghana), strong local NGO support facilitated SA implementation and was used to leverage more reluctant stakeholders; changes in local NGO interest or leadership were perceived to have affected implementation efforts. Technical support from international organizations, such as the WHO, was perceived to facilitate implementation. In particular, support for the in-country scientific and ethical review processes that preceded the assessment fieldwork and engagement of SA implementers from other countries to share their experiences with stakeholders in countries planning to use the SA were found to be especially helpful. Establishing a central governmental organization to work in collaboration with both local and international organizations to coordinate SA activities was also perceived to have facilitated implementation.

#### Countries’ ability to secure funding for all stages of SA implementation

Although all 15 countries secured funding to initiate the SA and conduct Stage 1 activities (i.e., strategic assessment fieldwork), countries varied in their opportunities to mobilize resources for the activities in subsequent stages. Knowledge transfer and exchange of information between countries on the implementation of Stage 2 interventions was perceived to have helped mobilize resources for Stage 3. However, many countries did not consistently monitor, evaluate, or report on the implementation of Stage 2 interventions. As a result, there was limited data on whether Stage 2 interventions were effective; this was identified as a potential barrier to scaling up these interventions.

#### Changes in the political climate during SA implementation

Changes in the political climate affected progression through SA stages. In Macedonia, for example, the political party that came to power following Stage 1 activities promoted aggressive, anti-abortion messages and changed the abortion law to make it more restrictive, thereby making it difficult to implement subsequent SA activities. An interview participant who provided technical assistance to a number of different countries mentioned how staff turnover resulting from political changes also affected SA implementation:

*‘I have been in countries where over a course of five years they had six different Ministers of Health, so that kind of change at that level often has knock down effects at lower levels in the ministries in changing priorities’. – Interview Participant # 118.*

#### Implementation of SA Stage 1: strategic assessment to identify needs and generate consensus recommendations to address needs

All 15 countries completed the Stage 1 strategic assessment activities in a similar manner with small logistical variations (e.g., size and composition of assessment team and coverage of assessment areas). Representatives from ministries, NGOs, professional associations, and national and international health care organizations participated in the following:

- Preparation of a background paper on existing socio-demographic, cultural, political, economic, and public health issues and available research on unintended pregnancy and abortion in the country;
- An assessment planning workshop using evidence from the background paper to develop strategic questions for guiding assessment activities, selecting and training assessment team members, and identifying assessment sites;
- Fieldwork involving iterative data generation through in-depth interviews and group discussions with a broad range of key informants to explore knowledge and perspectives of unintended pregnancy, contraception, abortion rights, and strategies for addressing SRH issues in the country. Data were analyzed and used to draft recommendations for specific follow-up actions; and
- A national dissemination meeting to present assessment findings to multiple stakeholders, such as policymakers, program managers, health care providers (HCPs), NGOs, UN agencies, SRH advocates, and local human rights organizations) who worked to generate consensus on the follow-up recommendations. See Additional file 8 for examples of Stage 1 recommendations commonly identified across countries.

Table 2 outlines the activities of Stage 1 and factors influencing Stage 1 implementation organized by SEM level (i.e., individual, community, organizational, and national/policy).

**Table 2:** Stage 1 activities and SEM factors influencing Stage 1 implementation

Stage 1 activities	SEM level factors influencing Stage 1 implementation
• Highlighted important issues related to unintended pregnancy and unsafe abortion and its impact on maternal deaths. • Generated consensus on follow-up recommendations for reforming legislation and policies to improve access to and quality of SRH services, improving provider-level capacity, and increasing community-level education regarding SRH services.	• Individual/Community/Organizational: Country’s ability to establish a cross-sectoral, multidisciplinary assessment team and ensure collaboration among team members with diverging perspectives. • Policy: Logistics of conducting strategic assessment fieldwork due to country size, geography, and language. • Individual: HCPs’ and community members’ buy-in and willingness to participate in assessment interviews/discussions. • Individual: Country stakeholders’ ability to reach consensus on recommendations to address assessment findings despite having different ideas about potential solutions; ○ E.g., some stakeholders perceived that all abortions were unsafe and that the solution was to increase access to contraception; others felt that they needed to increase access to safe abortion services. • Organizational: The WHO’s support helped ensure that power differentials among country stakeholders did not inhibit open dialogue and equitable participation during the consensus process.

#### Implementation of SA Stage 2: developing and testing innovations based on Stage 1 recommendations

The focus of Stage 2 activities varied among countries based on the legal status of abortion at the time of SA implementation; specifically whether they had restrictive abortion laws (e.g., Senegal, Sierra Leone and Malawi) or less restrictive abortion laws (e.g., Ghana, Kyrgyzstan, Moldova, Ukraine and Zambia). Table 3 outlines the Stage 2 activities and factors influencing Stage 2 implementation.

**Table 3:** Stage 2 activities and SEM factors influencing Stage 2 implementation

Stage 2 activities	SEM level factors influencing Stage 2 implementation
Countries with restrictive abortion laws	
• Advocated for legal reform to make abortion services legal and accessible. • Conducted quantitative research to better understand the magnitude of unsafe abortion and possible implications of liberalizing the law.	• Policy: Alignment of Stage 2 activities with country’s existing policy reform initiatives. ○ In Malawi and Sierra Leone, using the SA in conjunction with ongoing national discussions on liberalizing the law allowed the SA team to build on the groundwork initiated by advocates on these issues and garner their support for SA activities. • Community: Alignment of Stage 2 activities with community advocacy groups’ objectives. ○ In Malawi, the establishment and mobilization of community advocacy groups, such as the Coalition for Prevention of Unsafe Abortion, helped maintain pressure to influence policy reform.
Countries with less restrictive abortion laws	
• Piloted innovations to improve access to and quality of family planning, comprehensive abortion care (CAC), and post-abortion care (PAC) services. ○ Ghana, Macedonia, Moldova, Romania, Russia, Ukraine, and Zambia developed national standards and introduced technical guidelines. ○ Ghana, Kyrgyzstan, Moldova, Russia, and Sierra Leone trained HCPs to implement new or existing guidelines. ○ In Mongolia, Moldova, and Ukraine, a model of CAC was established and tested at specific intervention sites called “model clinics” to demonstrate high-quality CAC services and train HCPs. ○ Romania, Kyrgyzstan, and Zambia pilot tested services for different geographical and socioeconomic groups. • Raised awareness of the provisions of the law. ○ Ghana, Zambia, and Bangladesh conducted community-level sensitization workshops to reduce stigma around abortion and disseminated information on reproductive rights.	• Policy: Alignment of Stage 2 activities with country’s existing initiatives. ○ In Ukraine, dissemination of Stage 1 outputs aligned with the country’s concurrent efforts to update national abortion standards, which helped inform the development of these standards and obtain buy-in for their adoption. • Organizational: Alignment of Stage 2 activities with the mandate and areas of expertise of external organizations providing financial support. ○ In Zambia, an NGO’s interest in operations research led to the introduction of manual vacuum aspiration at pilot sites while WHO provided support for clinical research and guideline development. • Individual: HCPs’ personal views on providing contraceptive and abortion services. • Individual: HCPs’ knowledge and uptake of safe abortion laws, standards, and guidelines. • Organizational: Facility-level adoption of national safe abortion protocols and guidelines. • Policy: Availability of commodities (e.g., medical abortion drugs and equipment). • Individual/Community/Policy: Stigma and cost associated with accessing services.

#### Implementation of SA Stage 3: scaling up successful interventions

Stage 3 activities differed for each country depending on the progress made toward implementing Stage 2 activities and continued stakeholder buy-in. Although interview participants indicated that scale-up activities had been initiated in seven countries (i.e., Ghana, Moldova, Mongolia, Romania, Russian Federation, Ukraine, and Vietnam), there was limited information about the specific nature of these activities. Ukraine, Ghana, Moldova, and Romania initiated scale-up activities to expand access to family planning and safe abortion services beyond pilot sites. For example, Ukraine conducted a workshop to develop a scaling-up strategy for the Comprehensive Care for Unwanted Pregnancies project; the strategy was approved by the government and distributed to all regions for implementation. Table 4 outlines Stage 3 activities and factors influencing Stage 3 implementation.

**Table 4:** Stage 3 activities and SEM factors influencing Stage 3 implementation

Stage 3 activities	SEM level factors influencing Stage 3 implementation
• Limited data available on scaling up efforts.	• Consistent monitoring and evaluation of Stage 2 interventions to show effectiveness of the interventions. • Consistent reporting on Stage 2 interventions to inform scale up efforts. • Access to consistent technical support and funding for scaling up Stage 2 interventions.

### Perceptions of SA implementation

Interview participants’ descriptions of how the SA was implemented in their settings and perceptions of the key strengths of the SA directly aligned with the essential features or guiding principles of the approach itself: country-driven, participatory, and staged process using a systems framework.

#### Country-driven process

Interview participants shared that the decision to use the SA was always country initiated and activities were largely country-driven. Stakeholders selected their area of focus (e.g., contraceptive introduction, assessment of SRH policies and programs, or assessment of abortion service delivery), and developed strategic questions based on their existing systems and structures. They led all stages of the fieldwork, from identifying the pool of participants to selecting sites to reflect regional, cultural, socio-economic, and programmatic differences. The opportunity to adapt the SA to the local context facilitated ownership and acceptance of the assessment process, findings, and recommendations by country stakeholders. Stage 2 activities were also locally driven, often by local NGOs. Although the WHO and other international partners provided technical support on various topics (e.g., best practices for guideline development), country stakeholders led the process of developing local solutions based on their expertise in local challenges.

#### Participatory process

The participatory, multidisciplinary, and multisectoral approach of the Stage 1 strategic assessment process was thought to have provided an opportunity to integrate perspectives of often disparate groups of stakeholders to develop a shared understanding of key issues, as described by the quote below:

*‘I think that’s very important… the sort of the multidisciplinary nature of it is really unusual and really valuable aspect of the process, because very often, people don’t talk to each other. And also, many of them never really have had the opportunity previously to go deeply into communities to look at these issues and to talk to communities and to really see what the issues are on the ground. So the multidisciplinary, multi-stakeholder, really participatory nature of the Stage 1 of the strategic assessment are really critical I think.’ – Interview Participant # 116*.

Participation in assessment activities helped establish networks for decision making on how to deal with an issue like unsafe abortion that is often affected by social, political, and economic considerations. Participation in Stage 1 also increased team members’ commitment to implementing recommendations in Stage 2. For example, in Romania, the participation of representatives from the MOH, National Health Insurance, and National College of Physicians as part of the Stage 1 assessment team led to piloting a free contraceptives intervention for vulnerable groups in three districts (Stage 2) and scaling it up to 42 districts (Stage 3) in a relatively short period of time. To date, the Romanian government continues to allocate funds to this program every year.

#### Brings attention to important issues

Interview participants perceived the SA to have played an important role in creating dialogue and drawing attention to a sensitive and often taboo topic, as described in the quote below:

*‘Even for the program managers at the Ministry of Health who are involved in the strategic assessment, when they go out and talk to people they start understanding, aha, this is happening. I thought it was only a problem of young university students, you know, other’s problem, there are a lot of stereotypes about abortion. But when they go to the street and talk to people, then hear it is just like from the horse’s mouth. They go to a rural area and people in the rural area tell them that yes, during the three months 2 women died because of you know, botched abortion and things like that, an eye opener’.- Interview Participant # 101.*

Stage 1 helped to dispel common misconceptions about abortion and allowed individuals and groups with decision-making authority to examine whether their country’s existing policies and services were responsive to community needs. In some cases, the assessment helped highlight important social issues; for example, in Zambia, the assessment brought attention to the important issue of violence against women, and the country has since developed guidelines and programs to address some of these issues.

#### Staged and systematic approach

A number of interview participants, particularly frontline implementers of the SA, perceived the approach to serve as a structured and systematic process for identifying important issues and driving key activities. Outputs of Stage 1 (e.g., consensus recommendations) helped country stakeholders prioritize what to do next and introduce change in discrete, manageable units. The staged, incremental process allowed countries to move cautiously towards adopting recommended activities. For example, the SA was used to implement the national health strategy in Moldova, where linking pilot testing with ongoing monitoring and evaluation activities provided an opportunity to see the CAC model in action and demonstrate how to follow standards for medical abortion and manual vacuum aspiration for training HCPs.

## Discussion

The SA is designed as a participatory approach within a systems framework to identify and mobilize resources and relationships existing within the health system, which affect the feasibility, prioritization, acceptability, effectiveness, and sustainability of SRH interventions. This evaluation yielded important insights into the processes and mechanisms by which the SA was used to address issues related to unintended pregnancy and unsafe abortion in 15 countries. It helped unpack the “black box” of implementation by providing insight into how the activities within each stage of the SA were carried out and the factors in the implementation context that helped and hindered the implementation process.

Gaining perspectives from individuals who were involved with implementing the SA in these countries helped identify the strengths of the SA (i.e., how it is a country-driven, participatory, and systematic process for identifying and addressing key SRH issues) as well as the limitations of the SA. Implementing the SA is a complex and time consuming process; it requires countries to critically assess their existing SRH policies, programs, and services; consider the contextual factors and stakeholder relationships that influence each of these policies, programs and services; and identify and reach consensus on priority areas to focus SRH improvement efforts. To progress through SA stages, countries need to obtain and sustain stakeholder commitment and secure financial support and technical assistance for navigating challenges related to specific activities in each stage. Although Stage 1 outlines specific strategic assessment activities for all countries to engage in, country stakeholders have less direction on how to proceed through subsequent stages (e.g., specific activities related to piloting and scaling up) due to the variation in the scope and range of Stage 2 activities by country.

### Key success factors and opportunities to enhance SA implementation

This evaluation identified key success factors for implementing the SA and opportunities to leverage these factors to enhance ongoing and future implementation efforts in varied settings. It also identified country stakeholders’ key role in developing, piloting, and scaling up policies and programs to address SRH issues in their respective countries. First, countries that made the most progress towards implementing the SA were successful in engaging and maintaining country stakeholder support beyond Stage 1 activities. Opportunities to sustain engagement of country stakeholders beyond Stage 1 include sharing resources on SRH guidelines and interventions, inviting them to share their insights on how to plan for sustainability as part of Stage 1 activities and building capacity on how to scale up interventions, by sharing tools such as EXPANDNET capability building resources [22]. Second, clarifying that Stage 1 of the SA is an opportunity to better understand unintended pregnancy and unsafe abortion rather than to promote abortion services enhanced country stakeholders’ buy-in to launch the SA. Third, in some countries, following up Stage 1 assessment activities (i.e., qualitative interviews and focus group discussions) with quantitative data collection offered a complementary method for providing evidence to strengthen stakeholder support for reforming policies and programs. Fourth, establishing a central coordinating body (e.g., the strategic assessment team) to coordinate SA activities was perceived to facilitate SA implementation. When establishing a central coordinating body, countries can consider assessing leadership buy-in [23] or using coordinated leadership and organizational development strategies [24] to improve leadership and organizational supports for implementing and sustaining SA interventions. Countries can also employ a contingency plan for personnel turnover through an ongoing process of training and feedback to establish a stable team [25]. Coordination efforts can be further strengthened by using Communication for Development (C4D) strategies [26] to comprehensively target each level of the SEM for achieving the behavioral and social change needed to implement the SA. Fifth, leveraging Stage 1 outputs to obtain support and funding for Stage 2 activities facilitated implementation. For example, coordinating and integrating efforts of local NGOs and research organizations interested in reproductive health issues helped maximize in-country technical capacity to implement and evaluate Stage 2 interventions. Securing technical assistance from the WHO and other international organizations early on helped countries avoid losing the momentum or enthusiasm generated during Stage 1. Sixth, countries that monitored and reported on SA interventions were able to use these data to show progress and secure additional funding. Incorporating a formal evaluation plan (including process and outcome metrics) and using SRH reporting tools, such as Programme Reporting Standards for SRH programmes [27], can help countries ensure consistent monitoring of process and outcomes.

### Study limitations

There are limitations to evaluating the process of implementing the SA. First, interviews were conducted with a relatively small group of individuals. The SA is a multi-year process, and in some cases, retrospectively evaluating the process meant that the stakeholders who were initially involved with SA activities were no longer involved with ongoing efforts. This may have limited the number of country stakeholders available to participate in the evaluation. Second, the study design and methods were influenced by considerations of feasibility in terms of available resources, number of country sites, and availability of information. For example, face-to-face engagement with country stakeholders may have encouraged more responsiveness than email contact and telephone interviews; however, the former was not feasible due to resource limitations. Language and communication barriers in cases where English was not a first language may have also affected stakeholder involvement with the evaluation. Third, there was considerable variation in the quantity of data available for each country, which may affect the generalizability of findings. In addition, information from documents was not always linked directly to the SA. Most country documents had limited descriptions of Stage 2 and 3 activities, and these were often not explicitly linked to Stage 1 recommendations. Fourth, the document review phase included English-language articles only, potentially limiting the review of related documents in other languages. The document review process also required interpretation by the study team, which may have led to an information bias, misclassification, or errors in linking activities to output variables. We note, however, that the data calibration exercises were done with good agreement, and the document review phase was followed by interviews, which enabled the study team to supplement findings by collecting richer insights on factors related to implementation process and context [28]. Finally, because bias may have been introduced due to self-reporting, findings may not be applicable to other implementers and their experiences. The study team designed interview questions to encourage participants’ retrospective reflections on the implementation process, and data from multiple informants was compared and synthesized, which likely helped reduce bias from memory. Conclusions were based on the consistency of data from multiple sources and triangulation of data aimed at increasing the rigour of the data collection and analysis process.

### Next steps

This study focused on how the SA was applied in 15 countries to address issues of unintended pregnancy and unsafe abortion. Although we could not directly assess the quality with which each country applied the SA, the alignment between the perceived strengths of the SA and the key elements of the SA (e.g., country-driven, participatory, and systematic) suggest that the SA has been implemented as intended in most countries. The study identified gaps in the implementation process that should be addressed as well as opportunities that could be leveraged by others who are considering using the SA to address SRH issues in their countries.

Future studies may consider conducting an outcome or impact evaluation using quantitative outcomes data along with process data to identify changes in health systems and outcomes as a result of applying the SA.

## Conclusion

The SA is a country-driven, participatory, and comprehensive process model for identifying and addressing SRH issues. This process evaluation of 15 countries that applied the SA to address unintended pregnancy and unsafe abortion demonstrated how implementation is influenced by interactions between individuals, evidence, and context. The evaluation identified contextual factors that affected implementation at multiple levels in each country using the SEM as an analytical framework. This afforded a deeper understanding of the key success factors that are required for planning and executing SA activities. These factors include continued stakeholder engagement, coordination, monitoring documentation, and technical and financial support for implementing the SA. Lessons learned from this evaluation can be used to optimize the continued use of the SA and to inform future applications of the SA. Findings can also be used to guide similar activities, programs, and policies to improve access to and the quality of SRH services.

## Additional files

Additional file 1: Overview of frameworks. (DOCX 164 kb)

Additional file 2: Literature search strategy. (DOCX 17 kb)

Additional file 3: Data abstraction template. (DOCX 21 kb)

Additional file 4: Interview guides. (DOCX 28 kb)

Additional file 5: Coding criteria for analyzing interview data. (DOCX 26 kb)

Additional file 6: Overview of data sources by country. (DOCX 18 kb)

Additional file 7: Document review data sources. (DOCX 37 kb)

Additional file 8: Examples of common SA Stage 1 recommendations. (DOCX 16 kb)

## Abbreviations

CAC: Comprehensive abortion care
D&C: Dilation and curettage
HCPs: Health care providers
ICPD: International Conference on Population and Development
MOH: Ministry of Health
NGO: Non-governmental organization
PAC: Post-abortion care
SA: Strategic Approach to strengthening sexual and reproductive health policies and programs
SEM: Social ecological model
SRH: Sexual and reproductive health
WHO: World Health Organization
