# Supplementary material for: Evaluating implementation of the World Health Organization’s Strategic Approach to strengthening sexual and reproductive health policies and programs to address unintended pregnancy and unsafe abortion
Source: Reprod Health. 2017 Nov 21;14:153. doi: 10.1186/s12978-017-0405-3 (PMC5697396; doi:10.1186/s12978-017-0405-3)
Supplement: Supplementary file 1 — Overview of frameworks. (DOCX 164 kb) [file 12978_2017_405_MOESM1_ESM.docx]

# Additional File 1. Overview of frameworks

**Systems framework guiding the Strategic Approach (SA)**

One of the principles guiding the SA is that it uses a systems framework (Figure S1) to call attention to the many critical factors that together affect the feasibility, acceptability, effectiveness and sustainability of actions to improve reproductive health. This systems framework guided the *development* of the SA.

Figure S1. Systems framework guiding the SA

*Reference: WHO. The WHO Strategic Approach to strengthening sexual and reproductive health policies and programmes. Geneva: WHO, 2007.*

**Social Ecological Model (SEM)**

The SEM is a theory-based framework for understanding the multifaceted and interactive effects of personal and environmental factors that determine behaviors, and for identifying behavioral and organizational leverage points and intermediaries for health promotion within organizations. There are five nested, hierarchical levels of the SEM: Individual, interpersonal, community, organizational, and policy/enabling environment (Figure S2). We used the SEM to analyze the contextual factors that affected the *implementation* of the SA.

Figure S2. The Social Ecological Model


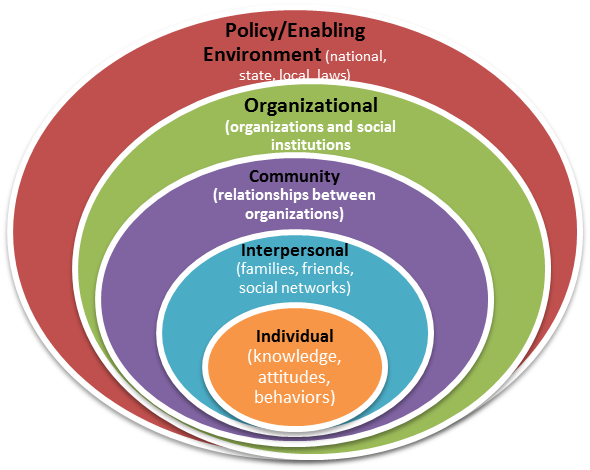


*Reference: Adapted from the Centers for Disease Control and Prevention (CDC), The Social Ecological Model: A Framework for Prevention, http://www.cdc.gov/violenceprevention/overview/social-ecologicalmodel.html (retrieved April 21, 2014).*

Definitions of SEM levels:

- Individual - Characteristics of an individual that influence behaviour change, including knowledge, attitudes, behavior, self-efficacy, developmental history, gender, age, religious identity, racial/ethnic identity, sexual orientation, economic status, financial resources, values, goals, expectations, literacy, stigma, and others.
- Interpersonal - Formal (and informal) social networks and social support systems that can influence individual behaviours, including family, friends, peers, co-workers, religious networks, customs or traditions.
- Community - Relationships among organizations, institutions, and informational networks within defined boundaries, including the built environment (e.g., parks), village associations, community leaders, businesses, and transportation.
- Organizational - Organizations or social institutions with rules and regulations for operations that affect how, or how well, for example, MNCH services are provided to an individual or group.
- Policy/Enabling Environment - Local, state, national and global laws and policies, including policies regarding the allocation of resources for maternal, newborn, and child health and access to healthcare services, restrictive policies (e.g., high fees or taxes for health services), or lack of policies that require childhood immunizations.
