# Supplementary material for: Evaluating implementation of the World Health Organization’s Strategic Approach to strengthening sexual and reproductive health policies and programs to address unintended pregnancy and unsafe abortion
Source: Reprod Health. 2017 Nov 21;14:153. doi: 10.1186/s12978-017-0405-3 (PMC5697396; doi:10.1186/s12978-017-0405-3)
Supplement: Supplementary file 2 — Literature search strategy. (DOCX 17 kb) [file 12978_2017_405_MOESM2_ESM.docx]

# Additional File 2. Literature search strategy

Database: Ovid MEDLINE(R) In-Process & Other Non-Indexed Citations and Ovid MEDLINE(R) <1946 to Present> Search Strategy:

1     "world health organization".mp. (99072)

2     ("W.H.O." adj health).ti. (32)

3     World Health Organization.mp. or "W.H.O.".au. (99074)

4     or/1-3 (99078)

5     (approach adj10 strategic).mp. (1512)

6     exp international cooperation/ (171842)

7     International organizations.mp. or exp health care organization/ (1191096)

8     internationality.mp. (56)

9     (intersectoral or inter-sectoral).mp. (1149)

10     "one world".mp. (183)

11     Action Plan.ti. (691)

12     exp public health service/ or exp public health/ or public health approach.mp. (181908)

13     or/5-12 (1402255)

14     Abortion.mp. or exp abortion/ (91115)

15     abort$.ti,ab. (66381)

16     birth control.mp. (5447)

17     contraception.ti. (10662)

18     Contraceptives.mp. or exp contraceptive agent/ (124116)

19     fertility control.mp. or exp birth control/ (165873)

20     exp family planning/ (32378)

21     induced abortion.mp. (18892)

22     (termination adj10 (fetal or foetal or fetus or foetus or labo$r or pregnancy or pregnant)).ti,ab. (9427)

23     pregnancy, unplanned/ (3072)

24     pregnancy, unwanted.mp. or exp unwanted pregnancy/ (2623)

25     (pregnancy adj5 (unwanted or unplanned)).mp. (6925)

26     or/14-25 (342185)

27     vietnam:.mp. or exp Vietnam/ (16247)

28     Mongolia:.mp. or Mongolia/ (7582)

29     ukrain:.mp. or exp ukraine/ (17055)

30     macedonia:.ti. or exp "Macedonia (Republic)"/ (1002)

31     Malawi:.mp. or Malawi/ (5416)

32     Zambia:.mp. or Zambia/ (4837)

33     Russia:.mp. or exp Russia/ (62975)

34     Ghan:.mp. or exp ghana/ (8529)

35     (senegal or seneg:).mp. or Senegal/ (8086)

36     Guinea:.mp. or Guinea/ (140364)

37     Kyrgyz:.mp. or exp Kyrgyzstan/ (1385)

38     sierra leon:.mp. or Sierra Leone/ (1508)

39     or/27-38 (270284)

40     13 and 26 and 39 (802)

41     4 and 26 and 39 (76)

List of databases searched:

| Medline search |
| --- |
| Gov Docs Library |
| Database Search [EMBASE](http://myaccess.library.utoronto.ca/login?url=http://gateway.ovid.com/autologin.html) |
| Database Search  [Columbia International Affairs Online (CIAO](http://www.ciaonet.org/) |
| Database Search [ProQuest](http://myaccess.library.utoronto.ca/login?url=http://search.proquest.com/advanced) |
| Database Search [PAIS International](http://myaccess.library.utoronto.ca/login?url=http://search.proquest.com/pais/advanced?accountid=14771) |
| Database Search Scopus |
| Database Search Web of Science |
| Grey Literature W.H.O. |
| Grey literature Individual Country Websites |
| Grey literature OECD |
| Grey literature [Google Custom Search (IGOs)](http://www.google.com/cse/home?cx=007843865286850066037:b0heuatvay8) |
| Grey literature [IGOs Search Engine](http://www.libraries.iub.edu/index.php?pageId=1000596) |
| Grey literature [Yearbook of International Organizations (online)](http://ybio.brillonline.nl.myaccess.library.utoronto.ca/s) |
| Grey Literature Custom search (NGOs) |
