# Supplementary material for: Evaluating implementation of the World Health Organization’s Strategic Approach to strengthening sexual and reproductive health policies and programs to address unintended pregnancy and unsafe abortion
Source: Reprod Health. 2017 Nov 21;14:153. doi: 10.1186/s12978-017-0405-3 (PMC5697396; doi:10.1186/s12978-017-0405-3)
Supplement: Supplementary file 3 — Data abstraction template. (DOCX 21 kb) [file 12978_2017_405_MOESM3_ESM.docx]

# Additional File 3. Data abstraction template

| Country Name | | | | Document Review Phase A | | | | Document Review Phase B | | | | | | | | | | |
| --- | --- | --- | --- | --- | --- | --- | --- | --- | --- | --- | --- | --- | --- | --- | --- | --- | --- | --- |
|  |  |  |  | Stage 1 (Assessment) | | | | Stage 2 (Developing/testing program innovations) | | | | | | Stage 3 (Scaling up successful interventions) | | | | |
| Section 1: Administrative Information | | | | | | | | | | | | | | | | | | |
| Document Number | | | |  | | | |  | | | | | |  | | | | |
| Document Year *(list)* | | | |  | | | |  | | | | | |  | | | | |
| Document Title *(list)* | | | |  | | | |  | | | | | |  | | | | |
| Author/Source *(full reference)* | | | |  | | | |  | | | | | |  | | | | |
| Type of Document *(e.g., country background paper, strategic assessment report, or  work plan.)* | | | |  | | | |  | | | | | |  | | | | |
| Reviewer Initials | | | |  | | | |  | | | | | |  | | | | |
| Date of Review | | | |  | | | |  | | | | | |  | | | | |
| Section 2: Recommendations | | | | | | | | | | | | | | | | | | |
| List of all recommendations | | | | # | | | | Recommendation | | | | | | | | | | |
|  |  |  |  |  | | | |  | | | | | | | | | | |
| Section 3: Description of Recommendations and Activities | | | | | | | | | | | | | | | | | | |
| Description Category | Applicable? | Implementation Stage | Cost | Care Stage | Target Audience | # | (Stage 1) | | (Stage 2) | Barriers | Facilitators | Dosage | Quality | | Responsiveness | Reach | Duration | (Stage 3) |
| Policies |  |  |  |  |  |  |  | |  |  |  |  |  | |  |  |  |  |
| Restrictions |  |  |  |  |  |  |  | |  |  |  |  |  | |  |  |  |  |
| Education |  |  |  |  |  |  |  | |  |  |  |  |  | |  |  |  |  |
| Persuasion |  |  |  |  |  |  |  | |  |  |  |  |  | |  |  |  |  |
| Incentivisation |  |  |  |  |  |  |  | |  |  |  |  |  | |  |  |  |  |
| Cooercion |  |  |  |  |  |  |  | |  |  |  |  |  | |  |  |  |  |
| Training |  |  |  |  |  |  |  | |  |  |  |  |  | |  |  |  |  |
| Enablement |  |  |  |  |  |  |  | |  |  |  |  |  | |  |  |  |  |
| Modelling |  |  |  |  |  |  |  | |  |  |  |  |  | |  |  |  |  |
| Environmental Restructuring |  |  |  |  |  |  |  | |  |  |  |  |  | |  |  |  |  |
| General |  |  |  |  |  |  |  | |  |  |  |  |  | |  |  |  |  |
| Section 4: Additional Reviewer Comments | | | | | | | | | | | | | | | | | | |
|  | | | | | | | | | | | | | | | | | | |
