# Supplementary material for: Evaluating implementation of the World Health Organization’s Strategic Approach to strengthening sexual and reproductive health policies and programs to address unintended pregnancy and unsafe abortion
Source: Reprod Health. 2017 Nov 21;14:153. doi: 10.1186/s12978-017-0405-3 (PMC5697396; doi:10.1186/s12978-017-0405-3)
Supplement: Supplementary file 4 — Interview guides. (DOCX 28 kb) [file 12978_2017_405_MOESM4_ESM.docx]

**Additional File 4. Interview guides**

| **Interview Guide - Frontline** | |
| --- | --- |
| **Instructions for facilitators:**   - *Welcome and introductions* - *Obtain verbal consent* - *Review process for interview* | |
| **Legend:**   - **Questions** and *Instructions* are indicated as such in the left hand column. *Instructions* are meant to be directions for the participants, given to them by the interviewer. Directions for the interviewer are indicated in *italics* in the body of the text of the second column. | |
| *Background* | The World Health Organization’s (WHO) *Strategic Approach to strengthening sexual and reproductive health policies and programs* (herein referred to collectively as “the SA”) is a three-stage planning, policy and program development process that includes:   - Stage 1: A field-based strategic assessment to identify and prioritise sexual and reproductive health needs, and generate consensus recommendations for addressing those needs and a broad stakeholder mandate for action; - Stage 2: Development, piloting-testing, monitoring and evaluation of policy, program and service-delivery interventions that address strategic assessment (i.e., Stage 1) recommendations; - Stage 3: Scaling up to expand access beyond model clinics, strengthen health system capacity to sustain the provision   This interview is being conducted as a part of a larger process evaluation to understand how the SA has been utilized in [insert country name] to date in the area of unintended pregnancy and unsafe abortion. |
| *Instructions* | The purpose of this interview is to explore your perceptions of the SA, and factors related to process and context that may have influenced the application of the SA at each stage. |
| **Question 1**  (Role in SA) | Please describe your role in relation to the SA. |
| **Question 2**  (SA Stage 1) | **The following questions relate to Stage 1 of the SA – the field-based strategic assessment to identify and prioritize sexual and reproductive health needs and generate recommendations for addressing those needs.**   - Please briefly describe the strategic assessment conducted in [insert country name]. - Please describe the assessment process. - Who was involved with the assessment? Who were the stakeholders? - What were the key outcomes of the assessment? - What were some of the priority recommendations developed for addressing the needs identified by the strategic assessment? |
| **Question 3**  (SA Stage 2) | **The following questions relate to Stage 2 of the SA – the development, pilot-testing, monitoring and evaluation of policy, program and service-delivery interventions to address the Stage 1 recommendations.**   - Please describe the nature of interventions developed and/or pilot-tested to address the Stage 1 recommendations in your country. - In your opinion, how well do these interventions address the strategic assessment recommendations? - In your opinion, do you think these interventions have been successful? Why or Why not? |
| **Question 4**  (SA Stage 3) | **The following questions relate to Stage 3 of the SA – Scaling up interventions to expand access and strengthening health system capacity to sustain the provision.**   - Have any of the interventions that you described been scaled up?   - **If so**, describe how. **If not**, what opportunities exist for scaling up implementation? - Has health system capacity been strengthened to sustain any of the interventions described?   - **If so**, describe ways in which the system has been strengthened. **If not**, what opportunities exist for ensuring sustainability? |
| **Question 5**  Barriers and Facilitators | **The following questions relate to barriers and facilitators to implementing the SA.**   - What were some of the **barriers or challenges** to implementing the SA? How were these barriers addressed?   Probes:   - What are some of the barriers or challenges at the systems level? (e.g., *funding, policy, health care structure, geography, current cultural and political climate, etc.)* - What are some of the barriers or challenges at the level of the health care provider? (*e.g., skills, attitudes/beliefs, leadership, interprofessional working climate, etc.)* - What are some of the barriers or challenges at the level of the patients and communities? (e.g., *cultural beliefs, health seeking behaviours, preferences for care, etc.)* - Are there any barriers unique to a particular SA stage: Stage 1 (strategic assessment), Stage 2 (implementing interventions) or Stage 3 (scaling up/sustaining interventions)? If so, please describe. - What are some of the **facilitators or opportunities** that could aid in the implementation of the SA?   Probes:   - What are some of the facilitators at the systems level? (e.g., *alignment with current initiatives, political turnover/opportunity, updating health training curricula, etc.)* - What are some of the facilitators at the level of the health care provider? (e.g., *champions at each clinical level, strong leadership, reward systems/positive reinforcement, training, etc.)* - What are some of the facilitators at the level of the patients and communities? (e.g., *cultural beliefs, health seeking behaviours, preferences for care, etc).* - Are there any facilitators unique to a particular SA stage: Stage 1 (strategic assessment), Stage 2 (implementing interventions) or Stage 3 (scaling up/sustaining interventions)? If so, please describe. |
| **Question 6**  Quality | In your opinion, how well has the SA been applied in your country to date? |
| **Question 7**  Monitoring and Evaluation | - Are any of these interventions being actively monitored? - **If so**, how? **If not**, why not? - Have any of these interventions been evaluated?   - **If so**, please describe the evaluation process and results. **If not**, are there plans to evaluate these interventions in the future? |
| **Question 8**  Participant Responsiveness | - In your opinion, how appropriate was the SA to your context? How appropriate were the related interventions? - How engaged were you in each stage (as applicable)? |
| **Question 9**  Adaptation | Please describe whether there were any changes or modifications made to the SA overall? To any particular stage of the SA?   - - **If so**, how has the SA been adapted to your context? |
| **Question 10** | - Is there anything else that you would like to add? - Do you know of any data sources that may provide pertinent information regarding SA implementation? |
| **Thank participant and wrap up** | |

| **Interview Guide (Technical)** | |
| --- | --- |
| **Instructions for facilitators:**   - *Welcome and introductions* - *Obtain verbal consent* - *Review process for interview* | |
| **Legend:**   - **Questions** and *Instructions* are indicated as such in the left hand column. *Instructions* are meant to be directions for the participants, given to them by the interviewer. - Directions for the interviewer are indicated in *italics* in the body of the text of the second column. | |
| *Background* | The World Health Organization’s (WHO) *Strategic Approach to strengthening sexual and reproductive health policies and programs* (herein referred to collectively as “the SA”) is a three-stage planning, policy and program development process that includes:   - Stage 1: A field-based strategic assessment to identify and prioritise sexual and reproductive health needs, and generate consensus recommendations for addressing those needs and a broad stakeholder mandate for action; - Stage 2: Development, piloting-testing, monitoring and evaluation of policy, program and service-delivery interventions that address strategic assessment (i.e., Stage 1) recommendations; - Stage 3: Scaling up to expand access beyond model clinics, strengthen health system capacity to sustain the provision   This interview is being conducted as a part of a larger process evaluation to understand how the SA has been utilized across the 15 participating countries to date in the area of unintended pregnancy and unsafe abortion. |
| *Instructions* | The purpose of this interview is to explore your perceptions of the SA, and factors related to process and context that may have influenced the application of the SA at each stage in each of the 15 participating countries. |
| **Question 1**  (Role in SA) | - Please describe your role in relation to the SA. |
| **Question 2**  (SA Stage 1) | **The following questions relate to Stage 1 of the SA – the field-based strategic assessment to identify and prioritize sexual and reproductive health needs and generate recommendations for addressing those needs.**   - Can you identify some of the **common elements** of the field-based strategic assessments conducted to identify and prioritize sexual and reproductive health needs in participating countries? - Can you identify some of the **differences** apparent from the field-based strategic assessments conducted in participating countries? Were there any case examples that stood out? - In countries where recommendations were developed for addressing the needs identified by the strategic assessment, what were some of the **common priority recommendations**? - Were there any **unique recommendations**? |
| **Question 3**  (SA Stage 2) | **The following questions relate to Stage 2 of the SA – the development, pilot-testing, monitoring and evaluation of policy, program and service-delivery interventions to address the Stage 1 recommendations.**   - Please describe the nature of interventions implemented in participating countries to address the Stage 1 recommendations. . - In your opinion, how well do these interventions address the strategic assessment recommendations? - Do you think these interventions have been successful? Why or Why not? |
| **Question 4**  (SA Stage 3) | **The following questions relate to Stage 3 of the SA – Scaling up interventions to expand access and strengthening health system capacity to sustain the provision.**   - Have any of the interventions you described above been scaled up? - **If so**, please describe. **If not**, what opportunities exist for scaling up? - Do you think that the health system capacity in any of the countries have been strengthened to sustain the interventions described above? - **If so**, describe ways in which the system has been strengthened. **If not**, what opportunities exist for ensuring sustainability? |
| **Question 5**  Barriers and Facilitators | **The following questions relate to anticipated or actual barriers and facilitators to implementing the SA.**   - - What were some of the **anticipated or actual** **barriers or challenges** to implementing the SA? How were these barriers addressed?   Probes:   - What were some of the barriers or challenges at the systems level? (e.g., *funding, policy, health care structure, geography, current cultural and political climate, etc.)* - What were some of the barriers or challenges at the level of the health care provider? (*e.g., skills, attitudes/beliefs, leadership, interprofessional working climate, etc.)* - What were some of the barriers or challenges at the level of the patients and communities? (e.g., *cultural beliefs, health seeking behaviours, preferences for care, etc.)*   - Were there any barriers unique to a particular SA stage: Stage 1 (strategic assessment), Stage 2 (implementing interventions) or Stage 3 (scaling up/sustaining interventions)? If so, please describe.   - What were some of the **anticipated or actual** **facilitators or opportunities** that could aid in the implementation of the SA?   Probes   - What were some of the facilitators at the systems level? (e.g., *alignment with current initiatives, political turnover/opportunity, updating health training curricula, etc.)* - What were some of the facilitators at the level of the health care provider? (e.g., *champions at each clinical level, strong leadership, reward systems/positive reinforcement, training, etc.)* - What were some of the facilitators at the level of the patients and communities? (e.g., *cultural beliefs, health seeking behaviours, preferences for care, etc).*   - Were there any facilitators unique to a particular SA stage: Stage 1 (strategic assessment), Stage 2 (implementing interventions) or Stage 3 (scaling up/sustaining interventions)? If so, please describe. |
| **Question 6**  Quality | **The following questions relate to the quality of policy, program and service delivery interventions.**  Please describe some of the general trends with regards to how well the SA has been applied in participating countries? Please highlight any examples that stood out.   - How well have interventions been implemented? - How well have interventions been scaled up? - How well have interventions been sustained? - How well have interventions been monitored? - How well have interventions been evaluated? |
| **Question 7**  Participant Responsiveness | - In your opinion, was the SA appropriate in the context of the countries in which it was implemented? - How engaged were the stakeholders in each of the participating countries? |
| **Question 8**  Adaptation | - Please describe whether there were any changes or modifications made to the SA overall? To any particular stage of the SA? - Please describe some of these modifications |
| **Question 9** | - Is there anything else that you would like to add? - Do you know of any data sources that may provide pertinent information regarding SA implementation? |
| **Thank participant and wrap up.** | |
