# Supplementary material for: Evaluating implementation of the World Health Organization’s Strategic Approach to strengthening sexual and reproductive health policies and programs to address unintended pregnancy and unsafe abortion
Source: Reprod Health. 2017 Nov 21;14:153. doi: 10.1186/s12978-017-0405-3 (PMC5697396; doi:10.1186/s12978-017-0405-3)
Supplement: Supplementary file 5 — Coding criteria for analyzing interview data. (DOCX 26 kb) [file 12978_2017_405_MOESM5_ESM.docx]

# Additional File 5. Coding criteria for analyzing interview data

| Code | | | Coding Criteria | Illustrative quotes |
| --- | --- | --- | --- | --- |
| 1. Country | | | - Any information about how the SA was conducted in a specific country was coded here. - There are 15 sub-nodes: one for each country involved in the process evaluation. - This code allows us to determine what country differences and similarities exist in the application of the SA. | “Ukraine, there was a lot of activities in improving quality of care of late abortion or second trimester abortion with external assessments from the Netherlands and it was… very appreciated and highly, highly praised by the country.” |
| 1. Socio Ecological Model (SEM) | | *2.0 General* | - This model addresses the complexities and interdependences between socioeconomic, cultural, political, environmental, organizational, psychological, and biological determinants of behaviour [22]. - There are a number of versions of the SEM, which use slightly different classification of these levels. For the purpose of this study, we used the individual, community, organizational, and policy level. - Any information related to how the SA was conducted at the SEM levels was coded here. - There are 4 sub-nodes: one for each SEM level. - This code allows us to understand how factors at the individual, community, organization, and policy level influences a country’s ability to implement the SA. | “Well, I’m sure funding is an issue – in terms of where the funding comes from. I mean I know, for instance, Ipas funded the strategic assessment in Zambia that I was part of but really how much are we relying on NGOs to fund these things? Obviously governments – for the most part – do not include money for this kind of stuff in their budgets and so it’s going to almost always need to come from donors of some sort and that’s, makes it difficult for it to be sustainable and usually these issues are low on the priority list for governments – even if you’re lucky you might get them to have a budget line for contraceptives but to actually have something in the budget about abortion is incredibly difficult, so I think a sustainable source of funding to do both the Stage 2 and Stage 3 activities, I can imagine can be quite challenging.” |
|  |  | *2.1 Individual* | - Any information related to the characteristics of an individual that influence behaviour change was coded in this sub-node. - Examples: knowledge, attitudes/beliefs, preferences for care, health seeking behaviour, socio-economic status, personal history, and change targeted at the individual level. | “When you have these groups on board like in Malawi, like in Zambia, the people who were heading the reproductive health units there, very active, highly involved, committed, so they facilitated the process. When you have somebody who is not clear about, who has personal issues who has personal beliefs about abortion, they may be, they can be a barrier. “ |
|  |  | *2.2 Community* | - Any information related to formal or informal social networks and social support systems that can influence individual behaviour was coded in this sub-node. - Examples: social norms, societal standards, social/religious groups, social climate, culture and change targeted at the community level. | “There are some specificities related to abortion because particularly when you get into the African countries where it is so heavily stigmatized, it’s much different than talking about abortion in Vietnam where, or Romania where one of the women said oh yeah we get an abortion on the way to the supermarket.” |
|  |  | *2.3 Organizational* | - Any information related to relationships among organizations, institutions, and informational networks within defined boundaries or rules and regulations within organizations or social institutions about quality of operations was coded in this sub-node. - Examples: organizational incentives, organizational policies, organizational priorities, organizational quality of care standards, facility level capacity and infrastructure and change targeted at organizational level. | “If they had been trained [to provide abortion services] it’s not uncommon from them to be relocated to other clinics where they don’t have the available equipment to be able to provide a service or to not have the support of their managers in the facilities to be able to provide the service.” |
|  |  | *2.4 Policy* | - Any information related to local, state, national and global laws/polices and change targeted at the policy level was coded in this sub-node. - Examples: national policies, laws, and legislation; health system priorities; political climate; national resource allocation and funding; government structure; national insurance policy; national health standards and policies and national education curricula. | “In Malawi the law needed to be changed ‘cause the law was very restrictive… so I think one of the key recommendations was really starting a process to look at legal reform because I think that was a critical piece.” |
|  |  | *2.5 Time* | Any information about change over time in the relation to when SA was being implemented was coded here. | “Stage 1, 2, 3 should be understood as stages that could take 10 or 15 years all together by the time you complete the assessment to the pilot projects and then begin to scale up and institutionalize and achieve sustainability. So that kind of time frame expectation should be understood that these things don’t happen in a space of a couple of years.” |
| 1. SA Stages | *3.0 General* | | - Any information about how the different stages of the SA were implemented was coded here. - There are 3 sub-nodes: one for each stage of the SA. - This code allows us to determine how each stage was carried out across countries. | “Well in my opinion looking at the outcomes, the SA was applied especially for stage 1 was very well done. And then the stage 2 we were lucky to have support…and we have been able to scale up nationwide” |
|  | *3.1 Stage 1* | | - Any information about how the field-based strategic assessment to identify and prioritize SRH needs was carried out or what findings and consensus recommendations were generated was coded in this sub-node. - There are 5 sub-nodes representing the process, findings, recommendations, barriers for moving to Stage 2, and miscellaneous factors related to Stage 1 | “First stage of the strategic assessment, you put together a team and the team may be, it’s anywhere from 10-20 people. So they are, they are a group of stakeholders in and of themselves. But then you identify stakeholders nationally and you bring these together to basically get them to provide guidance to the assessment.” |
|  | *3.2 Stage 2* | | - Any information about the development and pilot testing of interventions (i.e., policy, programs, and services) at different levels of the health system and evaluation of these interventions to determine if implementation is feasible, acceptable, effective, and sustainable in the particular context was coded in this sub-node. - There are eight sub-nodes representing different types of interventions, including policy, capacity building, community-level programs, service-delivery interventions, research, resources, and barriers to moving to Stage 3 and the miscellaneous factors related to Stage 2. | “To develop the model on comprehensive abortion care which, was adapted to the conditions of the situation of Moldova and to test this model on some intervention sites, which we call the model clinics.” |
|  | *3.3. Stage 3* | | - Any information about scaling up or initiating scale up to expand access beyond pilot sites and strengthening health system capacity to sustain the provision of programs and services was coded in this sub-node. | “We have just started with 2 model centres there and it’s expanded now to 6. And those 6 model services are actually providing about 41% of safe abortions that are done but you know other centres are coming on through policy interventions that the Ministry of Health has tried to make.” |
| 1. Factors Affecting the Application of the SA | | | - Any information about factors that either hindered or facilitated the implementation of the SA overall and progression through the SA stages was coded here. | “What can be implemented you know is dependent on  so many things, donor interests and what the partners  have expertise in basically, so most of the  interventions have come out of the health field I guess  although some have come out of human rights field as  well.” |
| 1. Lessons Learned of the SA approach | | | - Any information about strengths of using the SA or suggestions for improving the SA was coded here. - There are 2 sub-nodes representing the strengths of and suggestions for improvement for the SA. | “I think it was a very important tool to make a political decision and to implement a very important intervention, which lead to a decreased need for abortions.” |
| 1. Critical Elements for the SA | | | - Any information about essential factors without which the implementation of the SA would not be possible was coded here. | “The workshops as much as anything… the output of those workshops is really to craft a proposal to mobilize resources to do it as well because that’s another huge, part of the enabling part, is having resources, is having the financial resources to start the work and then as the work progresses if it’s successful then it, the fundraising tends to get easier if things are going well.” |
| 1. Adaptations to the SA | | | - Any information about changes made to how the SA was implemented from the original WHO model was coded here. | “Definitely there would be differences in you know implementing the strategic assessment in the 4 countries. But those differences might have been minor like you know I think like logistics, number of members, not substantively. So I don’t remember any change of the approach in substantive ways. Rather if there was any change…maybe in one country there were only 3 strategy questions, interrelated strategy questions, in another country there could have been 2, in another, 4...but otherwise overall there was no major change in the substance of the methodology.” |
| 1. Other | | | - Any information about how the SA was implemented in the identified 15 countries that was not captured in the above nodes was coded here. | “I did another assessment, it was not part of the strategic assessment but as a follow-up of the strategic assessment, we also have done health facility assessment on unsafe abortion through the health system, standards, it is already published, this was a follow-up of the strategic assessment.” |
