# Supplementary material for: Evaluating implementation of the World Health Organization’s Strategic Approach to strengthening sexual and reproductive health policies and programs to address unintended pregnancy and unsafe abortion
Source: Reprod Health. 2017 Nov 21;14:153. doi: 10.1186/s12978-017-0405-3 (PMC5697396; doi:10.1186/s12978-017-0405-3)
Supplement: Supplementary file 6 — Overview of data sources by country. (DOCX 18 kb) [file 12978_2017_405_MOESM6_ESM.docx]

**Additional File 6. Overview of data sources by country**
