# Supplementary material for: Evaluating implementation of the World Health Organization’s Strategic Approach to strengthening sexual and reproductive health policies and programs to address unintended pregnancy and unsafe abortion
Source: Reprod Health. 2017 Nov 21;14:153. doi: 10.1186/s12978-017-0405-3 (PMC5697396; doi:10.1186/s12978-017-0405-3)
Supplement: Supplementary file 7 — Document review data sources. (DOCX 37 kb) [file 12978_2017_405_MOESM7_ESM.docx]

**Additional File 7: Document review data sources**

**Strategic Assessment Reports**

1. Bacci A. Improving quality of care for mothers and newborn babies in the Republic of Kyrgyzstan: Assessment of quality of maternal and neonatal services at hospital and primary health care levels. Kyrgyzstan: 2012.
2. Chowdhury SYM, Moni D, Sarkar T. A Situation Analysis of the Bangladesh National Menstrual Regulation Programme. Bangladesh: Reproductive Health Alliance, 2003.
3. Division Sante de la Reproduction, Direction Natationale de la Sante Publique. Normes et procedures en sante de la reproduction composantes communes en sante de la reproduction. Guinea: Ministere de la Sante Republique de Guinee, 2009.
4. Division of Reproductive Health, Department of Health. Strategic Assessment of Unwanted Pregnancies and Unsafe Abortions in Senegal. Senegal: Ministry of Health and Prevention Republic of Senegal, 2011.
5. Fajans P, Ross G, World Health Organization. Abortion in Viet Nam: an assessment of policy, programme and research issues.1999.
6. Government of the Republic of Zambia Ministry of Health. Standards and Guidelines for reducing unsafe abortion morbidity and mortality in Zambia. Zambia: Ministry of Health, 2009.
7. Ministry of Health Malawi. A Strategic Assessment of Policies, Laws and Interventions. Malawi: WHO, 2012.
8. Horga M. Abortion and contraception in Romania. A strategic assessment of policy, programme and research issues. Romania: 2004.
9. Ministry of Health of the Republic of Moldova. Strategic Assessment of Policy, Quality of and Access to Contraception and Abortion Services in the Republic of Moldova. Moldova: Ministry of Health of the Republic of Moldova, 2006.
10. Ministry of Health of Ukraine. Abortion and Contraception in Ukraine. Strategic Assessment of Questions, Policy, Programmes and Research. Report and Recommendations. Ukraine: WHO, 2008.
11. Ministry of Health of the Russian Federation. Abortion and contraception in the Russian Federation. Strategic Assessment policies, programs and research. The report and recommendations. Russian Federation: WHO, 2009.
12. Public Health Institute of the Ministry of Health. A strategic assessment of policy, program and research issues related to reducing the recourse to abortion and improving the quality of care of abortion and family planning services in Mongolia. Ulaanbaatar, 2003.
13. Reproductive and Child Health Directorate. Unsafe Abortion in Sierra Leone: A Report of Community and Health System Assessments. Sierra Leone: Ministry of Health and Sanitation, 2013.
14. World Health Organization. Abortion and Abortion Care Services in Ghana. A Strategic Assessment of Policy, Programme and Research Issues. Ghana: WHO, 2005.
15. Republic of Macedonia Ministry of Health. Strategic Assessment of Policy, Quality and Access to Contraception and Abortion in the Republic of Macedonia. Macedonia: Republic Institute for Health Protection (RIHP), 2008.

**Documents from country stakeholders**

| 1. Aboagye PK. Applying the WHO Strategic Approach to Unsafe Abortion in Ghana. Ghana: Keeping Our Promise Conference, 2010. |
| --- |
| 1. Chowdhury SYM, Moni D. A Situation Analysis of the Menstrual Regulation Programme in Bangladesh. Reproductive Health Matters. 2004; 12(24 Supplement):95-104. |
| 1. Colbourn T, et al. Maternal mortality in Malawi, 1977-2012. BMJ. 2013; 3(e004150):doi:10.1136/bmjopen-2013-004150. |
| 1. Comendant R. Evaluating and mapping comprehensive abortion care in the Republic of Moldova: Report on project activities undertaken in the period of time May 2014 – April 2015. Moldova: World Health Organization and Reproductive Health Training Center, Chisinau, Republic of Moldova, 2015. |
| 1. Comendant R. Scaling up ambulatory comprehensive abortion care in two perinatal centres and a youth centre in Moldova. Moldova: World Health Organization and Reproductive Health Training Center, Chisinau, Republic of Moldova, 2010. |
| 1. Comendant R. Scaling up ambulatory comprehensive abortion care in two perinatal centres in Republic of Moldova: Report on project activities within the period of time April 2012 – March 2013. Moldova: World Health Organization and Reproductive Health Training Center, Chisinau, Republic of Moldova, 2013. |
| 1. Comendant R. Testing a Pilot Comprehensive Abortion Care Intervention in Moldova Report on project activities within the period of time: August 2008 – July 2009. Moldova: World Health Organization and Reproductive Health Training Center, Chisinau, Republic of Moldova, 2009. |
| 1. Department of Obstetrics and Gynaecology College of Medicine. Curriculum for Bachelor Degree in Clinical Obstetrics. Malawi: University of Malawi, 2013. |
| 1. Directorate General of Family Planning. Bangladesh National Menstrual Regulation Services Guidelines. Bangladesh: Ministry of Health and Family Welfare Peoples' Republic of Bangladesh, 2011. |
| 1. Dixon-Mueller R. Innovations in reproductive health care: menstrual regulation policies and programs in Bangladesh. Stud Fam Plann. 1988;19(3):129–140. |
| 1. Fajans P, Simmons R, Ghiron L. Helping Public Sector Health Systems Innovate: The Strategic Approach to Strengthening Reproductive Health Policies and Programs. American Journal of Public Health. 2006; 96(3):435-440. |
| 1. Gebreselassie H, Aboagye P, Asare G, Mitchelle EMH, Addy J. An Assessment of the Readiness to Provide Comprehensive Abortion Care in the Greater Accra, Eastern & Ashanti Regions of Ghana. Ghana. |
| 1. Ghana Health Service. 2006. Prevention and Management of Unsafe Abortion: Comprehensive Abortion Care Services Standards and Protocols. Accra: Ghana. Ghana Health Service. |
| 1. Government of Sierra Leone. The Safe Abortion Act. Sierra Leone: President and Members of Parliament, 2015. |
| 1. Health Education and Research Association. Report: National Dissemination Meeting on Results of High-Level Consultive Meeting (June, Brussells) on Reproductive Health Commodity Security. Macedonia: HERA, 2012. |
| 1. Ipas Zambia. Comprehensive Abortion Care for Young Women. A pilot project in Lusaka, Zambia. Zambia: Ipas, 2014. |
| 1. Ipas Zambia. Comprehensive Abortion Care for Young Women. Preliminary results from an Ipas Zambia pilot project with fully integrated youth participation. Zambia: Ipas, 2014. |
| 1. IPPF European Network. Key Factors Influencing Contraceptive Use in Eastern Europe and Central Asia. Macedonia: UNFPA, 2012. |
| 1. Jackson E, et al. Applying the WHO Strategic Approach to Unsafe Abortion: Experience in Five Sub-Saharan African Countries. Draft Manuscript. |
| 1. Jackson E, Johnson BR, Gebreselassie H, Kangaude GD, Mhango C. Related to Sexual and Reproductive Health and Abortion in Malawi. Reproductive Health Matters. 2011; 19(37):133-143. |
| 1. Johnson BR, Horga M, Fajans P. A Strategic Assessment of Abortion and Contraception in Romania. Reproductive Health Matters. 2004; 12(24 Supplement):184-194. |
| 1. Kalilani-Phiri L, Gebreselassie H, Levandowski BA, Kuchingale E, Kachale F, Kangaude G. The severity of abortion complications in Malawi. International Journal of Gynecology & Obstetrics. 2015 Feb 28;128(2):160-4. |
| 1. Kargbo, SAS. The Sierra Leone Strategic Assessment on Unwanted Pregnancies  and Unsafe Abortions. |
| 1. Kapito E, Kazembe A, Maluwa A, Malata A, Odland JO. Attitudes towards contraceptive use among schooling adolescents in Malawi. Journal of Research in Nursing and Midwifery (JRNM). 2012; 1(4):47-55. |
| 1. Levandowski BA, et al. Investigating social consequences of unwanted pregnancy and unsafe abortion in Malawi: The role of stigma. International Journal of Gynecology and Obstetrics. 2012; 118(Supplement 2):S167-S171. |
| 1. Lewandowski BA, et al. The Incidence of Induced Abortion in Malawi. International Perspectives on Sexual and Reproductive Health. 2013; 39(2):88-96. |
| 1. Malawi National Reproductive Health Service Delivery Guidelines. Summary of National Reproductive Health Strategy. Malawi: Republic of Malawi, 2010. |
| 1. McCaw-Binns A, Mullings J. Evidence generation and synthesis to improve family planning, prevent unsafe abortion and prevent and control sexually transmitted and reproductive tract infections. InHRP External Evaluation 2008–2012: advancing sexual and reproductive health 2013 (pp. 101-152). WHO, Geneva. |
| 1. Ministry of Health of Republic of Macedonia. Evaluation of national family programme and training needs assessment of health care providers in the area of SRH/FP in Republic of Macedonia. Macedonia: UNFPA Country Office, 2013. |
| 1. Ministry of Health of the Republic of Moldova. Assessing the Quality of Pregnancy Termination Services and Compliance with Safe Abortion Standards. Moldova: World Health Organization and Reproductive Health Training Center, Chisinau, Republic of Moldova, 2015. |
| 1. Ministry of Health of Ukraine. Comprehensive Care for Unwanted Pregnancy Project. Final Report, Phase 1, August 2009 - October 2011. Ukraine: WHO, 2011. |
| 1. Ministry of Health. Rulebook on the Content and the Manner of Counseling for the Pregnant Woman Prior to the Termination of Pregnancy. Macedonia: Official Gazzette of the Republic of Macedonia, 2014. |
| 1. Mladenovik B, Ninova KS, Jovanovski B, Vuckovska A. Knowledge, attitudes and practices of medical abortion among gynaecologists in Macedonia. XEPA. |
| 1. Moshin V, Comendant R, Baltag V, Stratilă M, Petrov V. Contraception and Abortion in Republic of Moldova . Moldova: Ministry of Health of the Republic of Moldova, 2005. |
| 1. National Centre for Standardization and Measurement. Comprehensive Abortion Care. Mongolia: Mongolian National Standard (MNS), 2004. |
| 1. NGO "Women health and Family Planning". Ukraine Abortion Legislation. Ukraine: WHFP, 2012. |
| 1. Official Gazzette of the Republic of Macedonia. Law on Termination of Pregnancy. Macedonia: Republic of Macedonia, 2013. |
| 1. Order of Minister of Health Care Ukraine. Clinical protocol "Comprehensive care of unwanted pregnancy". Ukraine: Order of Minister of Health Care Ukraine, 2010. |
| 1. Order of Minister of Health Care Ukraine. Procedure. Providing comprehensive medical care to the pregnant woman during unwanted pregnancy. Ukraine: Order of Minister of Health Care Ukraine, 2013. |
| 1. Rai RK, Singh PK, Kumar C, Singh L. Factors associated with the utilization of maternal health care services among adolescent women in Malawi. Home health care services quarterly. 2013 Apr 1;32(2):106-25. |
| 1. Republic of Macedonia Ministry of Health. National Strategy And Action Plan For Sexual And Reproductive Health In The Republic Of Macedonia By 2020. Macedonia: Public Health Institute of the Republic of Macedonia, 2010. |
| 1. Republic of Malawi Ministry of Health. Malawi 2010 EmONC Needs Assessment Final Report. Malawi: UNICEF, UNFPA, WHO, AMDD, 2011. |
| 1. Republic of Malawi Ministry of Health. Malawi National Reproductive Health Service Delivery Guideliens. Malawi: USAID, JHPIEGO, 2007. |
| 1. Republic of Malawi Ministry of Health. National Reproductive Health Strategy 2006-2010. Malawi: USAID, JHPIEGO, 2006. |
| 1. Republic of Sierra Leone Ministry of Health and Sanitation. Unsafe Abortion in Sierra Leone: A Report of Community and Health System Assessments Complete Report. Sierra Leone: Ipas, 2013. |
| 1. Republic of Zambia. Strategic Assessment of Policies, Programs and Research Issues Related To Prevention Of Unsafe Abortion In Zambia. Zambia: Ministry of Health Zambia, 2009. |
| 1. Talens AM, Willems FE. Second trimester abortion. Assessment report on second trimester abortion in Ukraine. Ukraine: WHO, 2013. |
| 1. Tsogt B, et al. Applying the WHO Strategic Approach to Strengthening. First and Second Trimester Abortion Services in Mongolia. Reproductive Health Matters. 2008; 16(31 Supplement):127-134. |
| 1. UNDP/UNFPA/WHO/World Bank Special Programme of Research, Development and Research Training in Human Reproduction (HRP). Abortion and Contraception in Romania. A Strategic Assessment of Policy, Programme and Research Issues. Romania: WHO, 2004. |
| 1. UNDP/UNFPA/WHO/World Bank Special Programme of Research, Development and Research Training in Human Reproduction (HRP). Abortion in Vietnam: An Assessment of Policy, Programme and Research Issues . Vietnam: WHO, 1999. |
| 1. UNDP/UNFPA/WHO/World Bank Special Programme of Research, Development and Research Training in Human Reproduction (HRP). Improving quality of care for mothers and newborn babies in the Republic of Kyrgyzstan. Assessment of quality of maternal and neonatal services at hospital and primary health care levels. Kyrgyzstan: WHO, 2012. |
| 1. UNDP/UNFPA/WHO/World Bank Special Programme of Research, Development and Research Training in Human Reproduction (HRP). Scaling up ambulatory comprehensive abortion care at three sites in Moldova: Final Report to The James Tudor Foundation. Moldova: WHO, 2016. |
| 1. Unit for Research in Reproductiv health in Guinea-Conakry. Plan d’action pour la reduction des avortements a risque en guinee apres l’evaluation strategique . Guinea: Cerreguicellule De Recherche En Sante De La Reproduction En Guinee, 2011. |
| 1. UNICEF and Ministry of Health of the Kyrgyz Republic. Maternal and Newborn Health in Chui Province & Kyrgyzstan: Assessment and Implications for Interventions. 2009. |
| 1. United Nations Population Fund. Family planning training for primary health care providers. Macedonia: UNFPA, 2014. |
| 1. United Nations Population Fund. Report of Reproductive Health Commodity Market Segmentation Research. Macedonia: UNFPA, 2013. |
| 1. WHO. Clinical practice guidelines for comprehensive abortion care. Malawi: WHO. |
| 1. WHO. Testing a Pilot Comprehensive Abortion Care Intervention in Moldova Final Report  on project activities within the period of time: September 2007 – December. Moldova: World Health Organization and Reproductive Health Training Center,  Chisinau, Republic of Moldova, 2007. |
| 1. World Health Organization. HRP Research Proposal: Training midwives in Kyrgyzstan to provide safe abortion care with mifepristone and misoprostol. Kyrgyzstan: WHO, 2013. |
| 1. World Health Organization. Scaling up health service delivery: from pilot innovations to policies and programmes. Vietnam: WHO, 2007. |
| 1. Zhylka NJ, Kvashenko VP, Slabkyj GA, Oshovskaja TT, Kashtaljan MM. Situation Review of the Quality of Family Planning Services and Reproductive Health. Basic Document.. Ukraine: WHO, 2007. |

**Documents from peer-reviewed literature**

1. Abdullah F, Troedsson H, Cherian M. The World Health Organization Program for Emergency Surgical, Obstetric, and Anesthetic Care. Archives of Surgery. 2011;146(5):620-3.
2. Anonymous. Research changes government policy in Zambia. Progress in human reproduction research. 1996(38):5-6.
3. Anonymous. Africa's unsafe abortions. Africa health. 1998;21(1):43.
4. Baiden F. Making safe abortion services accessible in Ghana. Journal of women's health (2002). 2009;18(12):1923-4.
5. Barnard S, Kim C, Park MH, Ngo TD. Doctors or mid-level providers for abortion. The Cochrane database of systematic reviews. 2015;7:CD011242.
6. Beck C, Berry NS, Choijil S. Health system reform and safe abortion: a case study of Mongolia. Global public health. 2013;8(2):174-86.
7. Berer M. Provision of abortion by mid-level providers: international policy, practice and perspectives. Bulletin of the World Health Organization. 2009;87(1):58-63.
8. Boonstra HD. Learning from adolescents to prevent HIV and unintended pregnancy. Issues in brief (Alan Guttmacher Institute). 2007:1-6.
9. Callister LC. Global maternal mortality: Contributing factors and strategies for change. MCN The American Journal of Maternal/Child Nursing. 2005;30(3):184-94.
10. Chong YS, Mattar CN. Mid-level providers: a safe solution for unsafe abortion. Lancet. 2006;368(9551):1939-40.
11. Dalton VK, Xu X, Mullan P, Danso KA, Kwawukume Y, Gyan K, et al. International family planning fellowship program: advanced training in family planning to reduce unsafe abortion. International perspectives on sexual and reproductive health. 2013;39(1):42-6.
12. D'Arcangues CM, Ba-Thike K, Say L. Expanding contraceptive choice in the developing world: Lessons from the Lao People's Democratic Republic and the Republic of Zambia. European Journal of Contraception and Reproductive Health Care. 2013;18(6):421-34.
13. Deniaud F. Update on the female condom in sub-Saharan Africa. Cahiers Sante. 1997;7(6):405-15.
14. Dikke G. Social factors determining the method of unwanted pregnancy termination in Russia. European Journal of Contraception and Reproductive Health Care. 2013;18:S205-S6.
15. Dikke G. Why is access to medical abortion limited in the Russian Federation? European Journal of Contraception and Reproductive Health Care. 2013;18:S59-S60.
16. Dikke G, Erofeeva L. Some errors after the medical abortion recovery period and contraception choice. European Journal of Contraception and Reproductive Health Care. 2012;17:S52.
17. Dikke G, Erofeeva L, Yarotskaya E, Nikolayeva E, Tsantsinger T. The vacuum-aspiration introduction into the ob\gyn out-patient practice: The role of training. European Journal of Contraception and Reproductive Health Care. 2014;19:S159-S60.
18. Dikke G, Yarotskaya E, Erofeeva L, Lazdane G, Kommendant R. Availability and restrictions of the medical abortion in Russia. European Journal of Contraception and Reproductive Health Care. 2010;15:71-2.
19. Dikke GB, Kochev DM. Errors in after medical abortion recovery period and their overcoming through the training of OB&Gyn. International Journal of Gynecology and Obstetrics. 2012;119:S327.
20. Erofeeva L. Demographic policy and reproductive rights-is consensus ever possible? European Journal of Contraception and Reproductive Health Care. 2013;18:S58-S9.
21. Eva G, Juana S, Huntington D. Evaluating family planning vouchers as a demand-side financing mechanism for marie stopes sierra leone. International Journal of Gynecology and Obstetrics. 2012;119:S579.
22. Ganatra B, Bygdeman M, Phan BT, Nguyen DV, Vu ML. From research to reality: The challenges of introducing medical abortion into service delivery in Vietnam. Reproductive Health Matters. 2004;12(24 SUPPL.):105-13.
23. Golota VI, Averina TA. [Ambulatory abortion, its advantages and economic effectiveness]. Ambulatornyi abort, ego preimushchestva i ekonomicheskaia effektivnost'. 1992(2):34-7.
24. Goyaux N, Alihonou E, Diadhiou F, Leke R, Thonneau PF. Complications of induced abortion and miscarriage in three African countries: a hospital-based study among WHO collaborating centers. Acta obstetricia et gynecologica Scandinavica. 2001;80(6):568-73.
25. Grove D, Hooper DJ. Doctor contraceptive-prescribing behaviour and women's attitudes towards contraception: two European surveys. Journal of Evaluation in Clinical Practice. 2011;17(3):493-502.
26. Gurina NA, Vangen S, Forsén L, Sundby J. Maternal mortality in St. Petersburg, Russian Federation. Bulletin of the World Health Organization. 2006;84(4):283-9.
27. Hainsworth G, Engel DMC, Simon C, Rahimtoola M, Ghiron LJ. Scale-up of adolescent contraceptive services: Lessons from a 5-country comparative analysis. Journal of Acquired Immune Deficiency Syndromes. 2014;66(SUPPL. 2):S200-S8.
28. Henshaw SK, Singh S, Haas T. The Incidence of Abortion Worldwide. International Family Planning Perspectives. 1999;25(9999):S30-S7.
29. Kodio B, De Bernis L, Ba M, Ronsmans C, Pison G, Etard JF. Levels and causes of maternal mortality in Senegal. Tropical Medicine and International Health. 2002;7(6):499-505.
30. Kuzmenko O, Rastrigina T. Building a sustainable reproductive health programme that contributes to the achievement of millennium development goals by investing in family planning interventions. European Journal of Contraception and Reproductive Health Care. 2013;18:S87.
31. Lazdane G. Regional experience of implementation of WHO tools and guidelines in the area of SRH. European Journal of Contraception and Reproductive Health Care. 2010;15:18-9.
32. Mbizvo M, Festin M. Successful family planning programmes in developing countries. European Journal of Contraception and Reproductive Health Care. 2013;18:S36-S7.
33. Ross JA, Pham SB. Unmet need for contraception in Vietnam: Who needs what and when. Social Biology. 1997;44(1-2):111-23.
34. Sefogah PE, Gurol I. Impact of free maternal care policy on maternal and child health indicators in Ghana. BJOG: An International Journal of Obstetrics and Gynaecology. 2015;122:14.
35. Shellenberg KM, Hessini L, Levandowski BA. Developing a Scale to Measure Stigmatizing Attitudes and Beliefs About Women Who Have Abortions: Results from Ghana and Zambia. Women & Health. 2014;54(7):599-616.
36. Simmons R, Fajans P. Contraceptive introduction reconsidered: A new methodology for policy and program development. Journal of Women's Health. 1999;8(2):163-73.
37. Simmons R, Hall P, Díaz J, Díaz M, Fajans P, Satia J. The strategic approach to contraceptive introduction. Studies in Family Planning. 1997;28(2):79-94.
38. Speciale AM, Solsona IPM. The use of mobile phones (mHealth) as a tool for reproductive health in Sub-Saharan Africa. Tropical Medicine and International Health. 2011;16:296.
39. Stanback J, Diabate F, Dieng T, de Morales TD, Cummings S, Traore M. Ruling out pregnancy among family planning clients: the impact of a checklist in three countries. Studies in family planning. 2005;36(4):311-5.
40. Tuyet HTD. Reduction maternal mortality rate in Viet nam: Comprehensive abortion care project. Journal of Perinatal Medicine. 2013;41.
41. Van Look PFAMDP, Cottingham JM. The World Health Organization's Safe Abortion Guidance Document. American Journal of Public Health. 2013;103(4):593-6.
42. Warriner IK, Meirik O, Hoffman M, Morroni C, Harries J, My Huong NT, et al. Rates of complication in first-trimester manual vacuum aspiration abortion done by doctors and mid-level providers in South Africa and Vietnam: a randomised controlled equivalence trial. Lancet. 2006;368(9551):1965-72.
43. Wesson J, Gmach R, Gazi R, Ashraf A, Mendez JF, Olenja J, et al. Provider views on the acceptability of an IUD checklist screening tool. Contraception. 2006;74(5):382-8.
44. Zaglada N, Moiseenko R. Putting program into action in Ukraine: Promoting Government investment in FP services for vulnerable populations. European Journal of Contraception and Reproductive Health Care. 2010;15:93-4.

**Documents from grey literature**

1. World Health Organization, & Population Council. (2007). Using the programme guidance tool to control RTIs in Ghana. Retrieved from World Health Organization website: <http://www.who.int/reproductivehealth/publications/rtis/ghana.pdf>
2. World Health Organization. (2007). The WHO strategic approach to strengthening sexual and reproductive health policies and programmes. Retrieved from World Health Organization website: http://apps.who.int/iris/bitstream/10665/69883/1/WHO_RHR_07.7_eng.pdf
3. World Health Organization. (2007). The WHO strategic approach to strengthening sexual and reproductive health policies and programmes. Retrieved from World Health Organization website: <http://www.who.int/reproductivehealth/topics/countries/strategic_approach/en/>
4. World Health Organization, & Population Council. (2009). A strategic approach to strengthening control of reproductive tract and sexually transmitted infections: use of the programme guidance tool. Retrieved from World Health Organization website: <http://apps.who.int/iris/bitstream/10665/44184/1/9789241598569_eng.pdf>
5. World Health Organization, & Department of Health and Research. (2012). Safe abortion: technical and policy guidance for health systems. Retrieved from World Health Organization website: <http://www.who.int/reproductivehealth/publications/unsafe_abortion/9789241548434/en/>
6. *Advancing sexual and reproductive health*. (2012).
7. *Family planning: the unfinished agenda.* (2006).
8. World Health Organization, & Department of Reproductive Health and Research. (2009). Department of Reproductive Health and Research (RHR) Partner brief. Retrieved from WorldHealth Organization website: <http://www.who.int/reproductivehealth/publications/general/rhr_09_02/en/>
9. World Health Organization, Department of Reproductive Health and Research including UNDP/UNFPA/WHO/World Bank Special Programme of Research, Development and Research Training in Human Reproduction. (2009). Biennial technical report 2007-2008. Retrieved from World Health Organization website: <http://www.who.int/reproductivehealth/publications/reports/9789241598309/en/>
10. UNDP/UNFPA/WHO/World Bank Special Programme of Research, & Development and Research Training in Human Reproduction (HRP). (2009). Highlights of 2008 - UNDP/UNFPA/WHO/World Bank Special Programme of Research, Development and Research Training in Human Reproduction (HRP). Retrieved from World Health Organization website: <http://www.who.int/reproductivehealth/publications/general/rhr_09_09/en/>
11. World Health Organization, & Department of Reproductive Health and Research. (2009). Highlights of 2008 – Reproductive Health and Research (RHR). Retrieved from World Health Organization website: <http://www.who.int/reproductivehealth/publications/general/rhr_09_10/en/>
12. World Health Organization, & Department of Reproductive Health and Research. (2010). Progress report - reproductive health strategy to accelerate progress towards the attainment of international development goals and targets. Retrieved from World Health Organization website: <http://www.who.int/reproductivehealth/publications/general/rhr_10_14/en/>
13. World Health Organization, & Department of Reproductive Health and Research. (2011). Highlights of 2010 UNDP/UNFPA/WHO/World Bank Special programme of research, development and research training in human reproduction (HRP). Retrieved from World Health Organization website: <http://www.who.int/reproductivehealth/publications/reports/rhr_hrp_11_20/en/>
14. World Health Organization. (2011). Biennial technical report 2009–2010 UNDP/UNFPA/WHO/World Bank Special Programme of Research, Development and Research Training in Human Reproduction (HRP). Retrieved from World Health Organization website: <http://www.who.int/reproductivehealth/publications/reports/9789241501743/en/>
15. World Health Organization, & Department of Reproductive Health and Research. (2012). Annual technical report 2011 Department of Reproductive Health and Research *including* UNDP/UNFPA/WHO/World Bank Special Programme of Research, Development and Research Training in Human Reproduction. Retrieved from World Health Organization website: <http://www.who.int/reproductivehealth/publications/reports/rhr_12_12/en/>
16. World Health Organization. (2013). Highlights of 2012 Department of Reproductive Health and Research *including* UNDP/UNFPA/UNICEF/WHO/World Bank Special Programme of Research, Development and Research Training in Human Reproduction. Retrieved from World Health Organization website: <http://www.who.int/reproductivehealth/publications/reports/hrp_1304/en/>
17. World Health Organization, & Department of Reproductive Health and Research. (2013). Annual technical report 2012 Department of Reproductive Health and Research *including* UNDP/UNFPA/UNICEF/WHO/World Bank Special Programme of Research, Development and Research Training in Human Reproduction. Retrieved from World Health Organization website: <http://www.who.int/reproductivehealth/publications/reports/rhr_1305/en/>
18. World Health Organization. (2014). Highlights of 2013 Department of Reproductive Health and Research *including* UNDP/UNFPA/UNICEF/WHO/World Bank Special Programme of Research, Development and Research Training in Human Reproduction. Retrieved from World Health Organization website: <http://www.who.int/reproductivehealth/publications/reports/highlights13/en/>
19. World Health Organization. (2014). Annual technical report 2013 Department of Reproductive Health and Research *including* UNDP/UNFPA/UNICEF/WHO/World Bank Special Programme of Research, Development and Research Training in Human Reproduction (HRP). Retrieved from World Health Organization website: <http://www.who.int/reproductivehealth/publications/reports/atr2013/en/>
20. World Health Organization. (2015). Highlights of 2014 Department of Reproductive Health and Research *including* UNDP/UNFPA/UNICEF/WHO/World Bank Special Programme of Research, Development and Research Training in Human Reproduction (HRP). Retrieved from World Health Organization website: <http://www.who.int/reproductivehealth/publications/reports/highlights2014/en/>
21. World Health Organization. (2015). Annual technical report 2014 Department of Reproductive Health and Research including UNDP/UNFPA/UNICEF/WHO/World Bank Special Programme of Research, Development and Research Training in Human Reproduction (HRP) . Retrieved from World Health Organization website: <http://www.who.int/reproductivehealth/publications/reports/atr2014/en/>
22. *External Evaluation 2008-2012.* (2013).
23. *Department of Reproductive Health and Research including UNDP/UNFPA/UNICEF/WHO/World Bank Special Programme of Research, Development and Research Training in Human Reproduction.* (All years). Retrieved from World Health Organization website: <http://www.who.int/reproductivehealth/about_us/en/>
24. *One Plan 2012 - 2016 Vietnam and the United Nations.* (2012). Retrieved from World Health Organization website: <http://www.who.int/country-cooperation/what-who-does/strategies-and-briefs/en/>
25. *WHO Abortion.* (1994-2014). Retrieved from World Health Organization website: [*http://www.who.int/reproductivehealth/publications/unsafe_abortion/en/*](http://www.who.int/reproductivehealth/publications/unsafe_abortion/en/)
