# Supplementary material for: Evaluating implementation of the World Health Organization’s Strategic Approach to strengthening sexual and reproductive health policies and programs to address unintended pregnancy and unsafe abortion
Source: Reprod Health. 2017 Nov 21;14:153. doi: 10.1186/s12978-017-0405-3 (PMC5697396; doi:10.1186/s12978-017-0405-3)
Supplement: Supplementary file 8 — Examples of common SA Stage 1 recommendations. (DOCX 16 kb) [file 12978_2017_405_MOESM8_ESM.docx]

**Additional File 8. Examples of common SA Stage 1 recommendations**

| **Policies** | Develop, disseminate, and implement national standards and protocols on comprehensive abortion care (CAC) aligning with WHO recommendations in all facilities offering abortion services. |
| --- | --- |
|  | Ensure informed consent, confidentiality, and privacy of women seeking abortion services. |
|  | Define criminal, illegal, and unsafe abortions in normative documents and implement a system of accountability for violators. |
|  | Harmonize laws in countries that have ratified international and regional human rights legal instruments. |
|  |  |
| **Services and resources** | Improve access to abortion services and emergency contraceptives and establish outpatient consultation centres for ambulatory CAC. |
|  | Increase budgetary allocation for contraceptive procurement and distribution. |
|  | Define and unify costs of services and ensure insurance coverage for socially disadvantaged women. |
|  | Increase availability of equipment for manual vacuum aspiration (MVA), medical abortion drugs (e.g., Mifepristone and Misoprostol), protective equipment (e.g., sterile gloves and soap) and antibiotics (e.g., Doxycycine) in all facilities providing abortions. |
|  |  |
| **Provider training and education** | Establish a national training curriculum for medical, midwifery, and nursing schools to standardize knowledge on stages of abortion procedure, medical abortion, supervision, post-abortion care (PAC), and contraception. |
|  | Include training on nontechnical aspects of abortion, such as patient interactions and social and psychological counselling. |
|  | Distribute information, education, and communication materials on CAC to all levels of the healthcare system. |
|  |  |
| **Monitoring and evaluation** | Institutionalize standardized system for collecting and recording statistical data to monitor and evaluate abortion services performed (e.g., methods, rates, women's age, mortality, and complications). |
|  | Design research studies to explore attitudes and behaviours around abortion and collect evidence on unsafe abortion and its contribution to maternal mortality. |
|  | Implement facility-level service quality monitoring system through provider self-assessment checklists and patient satisfaction questionnaires that measure quality indicators (e.g., abortion method, pain control, patient satisfaction, level of complications, and PAC). |
|  |  |
| **Community awareness** | Promote prevention of unintended pregnancy through mass media and sex education curricula targeted at primary, secondary, and tertiary school students; community members, teachers and traditional leaders; and HCPs. |
|  | Develop youth-friendly services and collaborate with education, psychological, and social assistance programs to consolidate a network of youth-friendly centres. |
